# A Positive Regulatory Loop between *foxi3a* and *foxi3b* Is Essential for Specification and Differentiation of Zebrafish Epidermal Ionocytes

**DOI:** 10.1371/journal.pone.0000302

**Published:** 2007-03-21

**Authors:** Chung-Der Hsiao, May-Su You, Ying-Jey Guh, Ming Ma, Yun-Jin Jiang, Pung-Pung Hwang

**Affiliations:** 1 Institute of Cellular and Organismic Biology, Academia Sinica, Taipei, Taiwan; 2 Institute of Molecular and Cell Biology, Singapore, Singapore; 3 Graduate Institute of Life Sciences, National Defense Medical Center, Taipei, Taiwan; Baylor College of Medicine, United States of America

## Abstract

**Background:**

Epidermal ionocytes play essential roles in the transepithelial transportation of ions, water, and acid-base balance in fish embryos before their branchial counterparts are fully functional. However, the mechanism controlling epidermal ionocyte specification and differentiation remains unknown.

**Methodology/Principal Findings:**

In zebrafish, we demonstrated that Delta-Notch-mediated lateral inhibition plays a vital role in singling out epidermal ionocyte progenitors from epidermal stem cells. The entire epidermal ionocyte domain of genetic mutants and morphants, which failed to transmit the DeltaC-Notch1a/Notch3 signal from sending cells (epidermal ionocytes) to receiving cells (epidermal stem cells), differentiates into epidermal ionocytes. The low Notch activity in epidermal ionocyte progenitors is permissive for activating winged helix/forkhead box transcription factors of foxi3a and foxi3b. Through gain- and loss-of-function assays, we show that the foxi3a-foxi3b regulatory loop functions as a master regulator to mediate a dual role of specifying epidermal ionocyte progenitors as well as of subsequently promoting differentiation of Na^+^,K^+^-ATPase-rich cells and H^+^-ATPase-rich cells in a concentration-dependent manner.

**Conclusions/Significance:**

This study provides a framework to show the molecular mechanism controlling epidermal ionocyte specification and differentiation in a low vertebrate for the first time. We propose that the positive regulatory loop between foxi3a and foxi3b not only drives early ionocyte differentiation but also prevents the complete blockage of ionocyte differentiation when the master regulator of foxi3 function is unilaterally compromised.

## Introduction

In terrestrial vertebrates, diverse types of ionocytes (ICs) that are distributed in the bladder, inner ear, and kidney have evolved to play essential roles in transepithelial ion, water, and acid-base transportation. For instance, ICs specializing in mediating salt and water absorption (principal cells), acid-base transport (intercalated cells), or both (inner medullary cells) have been characterized in collecting ducts of mouse kidneys [1], [2]. In the mouse inner ear, forkhead-related (FORE) cells are also reported to play an essential role in maintaining osmotic pressure and the ionic composition [3]. For aquatic vertebrates like fish, the maintenance of osmotic and ionic gradients between body fluids and the external environment for internal homeostasis are more challenging. Fish gills have evolved as the most important extra-renal organ to mediate ion transport and acid-base regulation for compensating the passive leakage of ions [4], [5]. At a cellular level, the functional units of ion and acid-base regulation in gills are carried out by branchial ICs (also known as chloride cells or mitochondrion-rich cells) which are primarily deposited on the junction regions between filaments and lamellae. At an organismic level, when the functional gill is not yet completely developed, epidermal ICs are precociously scattered on the skin to mediate ion and acid-base balance [6], [7], [8]. Based on morphology, vital dye binding and immunocytochemistry, distinct types of epidermal or branchial ICs have been reported in diverse fish species, and have been suggested to mediate an equivalent function as their counterparts in the kidneys of terrestrial vertebrates [9], [10]. However, very little is known about whether distinct types of ICs are generated from common progenitors and what the molecular mechanisms controlling IC specification and differentiation are. We noted that the slow progress of studying IC development in aquatic animals is largely caused by insufficient molecular and genetic tools for non-model fish. Therefore, we sought to use zebrafish as a new model to study IC development by taking advantage of a mature embryonic manipulation technique as well as many available genetic mutants [11], [12], [13]. Recently, our team identified two types of epidermal ICs as Na***^+^***,K***^+^***-ATPase-rich cells (NaRCs) and H***^+^***-ATPase-rich cells (HRCs) in zebrafish embryonic skin [14]. In this study, we extend our knowledge to the molecular mechanisms determining IC identity and promoting the differentiation of NaRCs and HRCs in zebrafish.

The Delta-Notch (D-N) lateral inhibition mechanism is an evolutionarily conserved signal transduction cascade and plays essential roles in cell fate choices and boundary formation [15]. Taking *Drosophila* sensory organ formation as an example, the position of the sensory organ precursor (SOP) is determined by the basic helix-loop-helix (bHLH) transcription factors of the Achaete-Scute complex (AS-C) [16]. Within the SOP cluster, a nascent SOP expresses cell surface DSL (Delta, Serrate, Lag-2) ligands, and activates Notch in neighboring cells. Activation of the Notch pathway results in proteolytic cleavage to release the intracellular domain of Notch (NICD), which is subsequently translocated into the nucleus and is associated with CSL (CBF1/Su(H)/Lag-1, also known as RBPjκ) proteins to activate target genes, such as the *Hairy/Enhancer of Split* (HES) family of bHLH transcriptional repressors (also known as bHLH-O). Activated bHLH-O inhibits the activity of *AS-C* genes in Notch signal-receiving cells (epidermal cells) and also prevents these cells from differentiating into SOPs [17], [18]. Failure to transmit the Delta-Notch lateral inhibition signal results in overproduction of SOPs in *Drosophila* [19]. In zebrafish embryos, the epidermal ICs are surrounded by keratinocytes which are arranged in a mosaic pattern. This observation suggests that epidermal IC cell fate choice might also be mediated by the Delta-Notch lateral inhibition mechanism. We tested this hypothesis by examining the epidermal IC development in several genetic mutants or morphants which have defects in the Delta-Notch signaling pathway. Results demonstrated that Delta-Notch-mediated lateral inhibition indeed plays a role in selecting epidermal IC progenitors from the epidermal stem cell (SC) pool in zebrafish.

Winged helix/forkhead box transcription factors encode a large class of nuclear DNA-binding factors and function as cell fate determinants or cell differentiation regulators [20], [21], [22], [23], [24]. Recent studies have shown that IC differentiation in the mammalian inner ear and kidney is controlled by a strikingly conserved *foxi1*-dependent mechanism. Gene knockout experiments demonstrated that in the absence of *foxi1*, IC progenitors in the endolymphatic duct and collecting duct fail to differentiate into FORE cells and intercalated cells which respectively resulted in deafness [3] or distal renal tubular acidosis [25]. This *foxi1*-mediated process of IC differentiation in the inner ear and urinary system of terrestrial vertebrates inspired us to postulate whether a similar mechanism is also utilized by aquatic vertebrates for controlling epidermal IC differentiation. In zebrafish, four members of *foxi* genes have been identified as being expressed in the inner ear and pharyngeal arch (*foxi1*) [26], in the chordamesoderm, retina, and pharyngeal arches (*foxi2*) [27], and in mucous cells (*foxi3a* and *foxi3b*, we have renamed *foxi3a*- and *foxi3b*-expressing cells as epidermal ICs in this study) [27]. In this study, we manipulate the function of *foxi3* and *foxi3b* by morpholino knockdown and mRNA misexpression to test whether they have a function in epidermal IC development. The data obtained from gain- and loss-of-function assays led us to discover a positive regulatory loop between *foxi3a* and *foxi3b* which is essential not only for acquiring IC identity but also for promoting IC differentiation.

## Results

### Na^+^,K^+^-ATAPase-rich Cells (NaRCs) Differentiate Earlier than H^+^-ATPase-rich Cells (HRCs)

**Figure 1:**
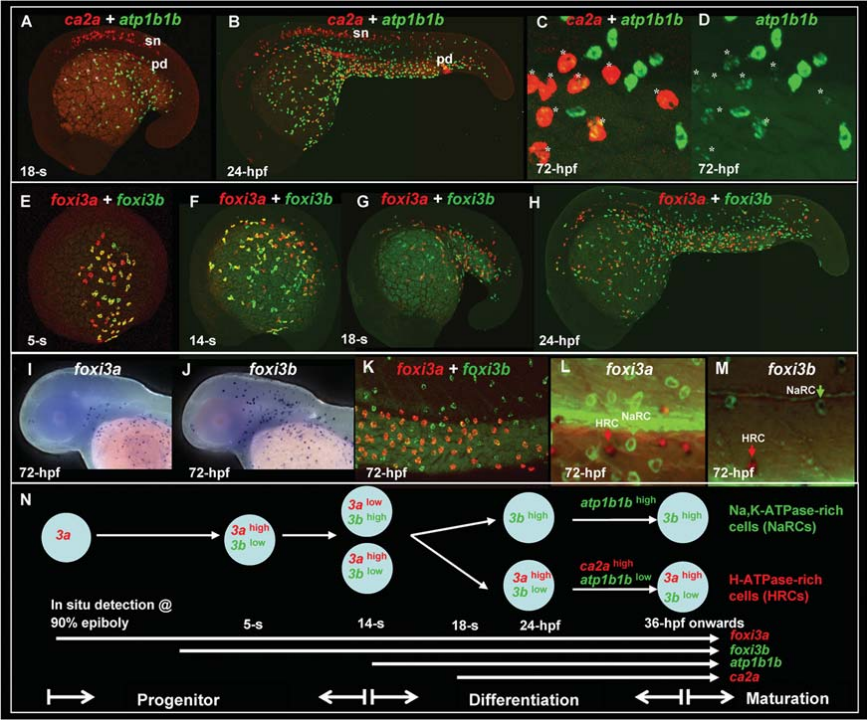
Early Development of Epidermal Ionocytes in Zebrafish Embryos. (A–D) Detection of *atp1b1b* (green) and *ca2a* (red) expression in differentiating epidermal ionocytes by fluorescent double *in situ* hybridization from the 18-somite (18-s) to 72-hour post fertilization (hpf) stage. Note that *ca2a* is also expressed on spinal cord neurons (sn) and the pronephric duct (pd) in A and B. In A, some epidermal ionocytes which were double-positive for both *ca2a* and *atp1b1b* are highlighted with asterisks. (C, D) High-magnification view of *ca2a* and *atp1b1b* expressions in the yolk extension region of 72-hpf embryos. Differentiating H^+^-ATPase rich-cells cells (HRCs) (labeled by asterisks) express a high level of *ca2a* and a low level of *atp1b1b,* while differentiating Na^+^,K^+^-ATPase-rich cells (NaRCs) are positive for only *atp1b1b*. (E–M) Dynamic expression of *foxi3a* (red) and *foxi3b* (green) in epidermal ionocyte progenitors and differentiating epidermal ionocytes from the 5-s to the 72-hpf stage. Whole-mount *in situ* hybridization of 72-hpf embryos show that the expression patterns between *foxi3a* (I) and *foxi3b* (J) are distinct in the cephalic domain. (K) In the yolk extension region, some cells express a high level of *foxi3a* (red) and a low level of *foxi3b* (green), while others are positive only for *foxi3b* (green). Immunodetection of Na^+^,K^+^-ATPase (green) and H^+^-ATPase (red) in 72-hpf embryos which were *in situ*-stained with either *foxi3a* (L) or *foxi3b* (M). Results showed that *foxi3a* was only expressed in HRCs (red arrow), while *foxi3b* was expressed by both HRCs (red arrow) and NaRCs (green arrow). (N) Schematic diagram showing the major events of epidermal ionocyte development in zebrafish embryos at the progenitor stage (the 90% epiboly to 14-s stages), differentiation stage (the 14-s stage to 36 hpf), and maturation stage (from 36 hpf onwards). The developmental stage is indicated in the left lower corner of each panel.

We have previously characterized two distinct populations of epidermal ICs, NaRCs and HRCs, in 3-day post-fertilization (dpf) zebrafish embryos which respectively expressed Na***^+^***,K***^+^***-ATPase and H***^+^***-ATPase in a mutually exclusive manner [14]. However, we failed to detect the early ontogeny of NaRCs and HRCs due to the low concentration of Na***^+^***,K***^+^***-ATPase and H***^+^***-ATPase which accumulated in epidermal ICs of embryos younger than 36 hours post-fertilization (hpf) (data not shown), indicating the difficulty in certifying the lineage relationships between NaRCs and HRCs by immunocytochemistry. To overcome this constraint, we tested several NaRC markers: the Na***^+^***,K***^+^***-ATPase α1a.2 subunit (*atp1a1a.2*), Na***^+^***,K***^+^***-ATPase α1a.4 subunit (*atp1a1a.4*), Na***^+^***,K***^+^***-ATPase α1a.5 subunit (*atp1a1a.5*), Na***^+^***,K***^+^***-ATPase β1b subunit (*atp1b1b*), N-myc downstream-regulated gene 1 (*ndrg1*), and potassium inwardly-rectifying channel, subfamily J, member 1 (*kcnj1*), as well as HRC markers: the H***^+^***-ATPase V0c subunit (*atp6v0c*), type II arginase (*arg2*), transient receptor potential cation channel, subfamily M, member 7 (*trpm7*), and carbonic anhydrase 2a (*ca2a*) by *in situ* hybridization. Among these epidermal IC markers, *atp1b1b* and *ca2a* were identified as being robust and early markers for labeling NaRCs and HRCs, respectively (*atp1b1b* is also weakly expressed in the differentiating HRC, see below for discussion). By performing fluorescent double *in situ* hybridization, we found that *atp1b1b* was expressed on epidermal ICs with a sporadic pattern as early as 14 hpf (data not shown). By the 18-somite (18-s) stage, *ca2a* was detected in about 10% of *atp1b1b*-positive cells (ure 1A, highlighted by asterisks). By 24 hpf, the differentiating NaRCs and HRCs had gradually spread out and were scattered on the epidermal layer in a pattern similar to those detected by antibody staining in 3-dpf embryos [14]: differentiating HRCs were largely distributed on the yolk sac and extension regions, while differentiating NaRCs were more widely scattered on the epidermis (Figure 1B). Interestingly, neither NaRCs nor HRCs ever appeared on the cephalic epidermis (Figure 1A and 1B). For 24-hpf embryos, the differentiating HRCs expressed a balanced level of *atp1b1b* and *ca2a* (Figure 1B). However, as development proceeded, the *atp1b1b* level in differentiating HRCs was gradually downregulated and maintained at a basal expression level by 72 hpf (Figure 1C and 1D, labeled by asterisks). Differentiating NaRCs, on the contrary, strongly expressed *atp1b1b* but lacked *ca2a* expression. This observation suggests that both NaRCs (*atp1b1b*-positive cells) and HRCs (*ca2a*-positive cells) might come from common progenitors and then subsequently differentiate.

### *foxi3a* and *foxi3b* are Early Markers for the Epidermal IC Progenitors

**Figure 2:**
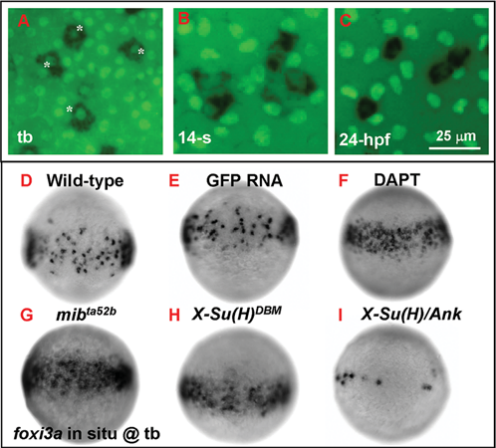
Number of Epidermal Ionocyte Progenitors is Restricted by Delta-Notch-Mediated Lateral Inhibition. (A–C) Dynamic expression of the epidermal ionocyte progenitor marker of *foxi3a* (black, cytoplasmic staining) and the epidermal stem cell marker of P63 (green, nuclear staining) on the zebrafish epidermis. In the beginning, *foxi3a* was activated in some P63-positive epidermal stem cells at the tail bud (tb) stage (indicated by asterisks, A). However, as development precedes, P63 expression in epidermal ionocyte progenitors is sharply downregulated (at the 14-somite (14-s) stage, B) ultimately to an undetectable level (24 hour post infection (hpf), C). (D–I) The mosaic distribution of epidermal ionocyte progenitors within the epidermal ionocyte domain is controlled by Delta-Notch-mediated lateral inhibition. In DAPT-treated embryos (F), and *mib^ta52b^*- (G) or *X-Su(H)^DBM^* mRNA-injected embryos (H), the *foxi3a* expression in the epidermal ionocyte domain has become denser and more homogeneous. While in *X-Su(H)/Ank* mRNA-injected embryos (I), *foxi3a* expression in the epidermal ionocyte domain is greatly reduced. The developmental stage is indicated in the left lower corner of each panel.

To test the common progenitor hypothesis, we needed a molecular marker to trace the early epidermal IC development before the onset of differentiation. *f*o*xi3a* and *foxi3b* are duplicated winged helix/forkhead transcription factors and are sporadically expressed on the epidermis at the gastrula stage [27]. According to fluorescent double *in situ* hybridization, *foxi3a* was expressed within a subdomain (hereafter, defined as the epidermal IC domain) of the ventral ectoderm at around the 90% epiboly to tail bud (tb) stages (Figure 2A). By the 5-s stage, *foxi3b* was rapidly upregulated in the majority of *foxi3a*-positive cells and resulted in a mixed population of cells with either *foxi3a* ^high^ (red), *foxi3a* ^high^/*foxi3b* ^low^ (yellow), or *foxi3a* ^low^/*foxi3b* ^high^ (light green) expression (Figure 1E). By the 14-s stage, *foxi3b* expression in *foxi3a*-positive cells was sharply upregulated, resulting in more cells showing *foxi3a* ^low^/*foxi3b* ^high^ (green) expression (Figure 1F). It is worth noting that by the 14-s stage, *foxi3a* expression in some *foxi3a* ^low^/*foxi3b* ^high^ cells was sharply downregulated to a very low level, suggesting that these cells were differentiating into different cell fates. From the 18-s stage onwards, the differentiation process may have been completed since the majority of cells expressed either *foxi3b* ^high^ or *foxi3a* ^high^/*foxi3b* ^low^ (Figure 1G and 1H). By 24 hpf, we noted that the spatial distribution patterns of *foxi3a* and *foxi3b* were highly similar to those of HRCs and NaRCs (compare Figure 1B with 1H or [14]). The comparable distribution pattern persisted until at least 72 hpf (Figure 1I and 1J). *foxi3a*-positive cells had a restricted distribution on the yolk and the yolk extension region (similar to the *ca2a* expression pattern); while *foxi3b*-positive cells were widely scattered on the epidermis (similar to the *atp1b1b* expression pattern). In addition, we also noted that the colocalized expressions of *foxi3a* and *foxi3b* (*foxi3a* ^high^ */foxi3b* ^low^) in the yolk extension region (Figure 1K) were highly similar to those of *ca2a* and *atp1b1b* (Figure 1C). To clarify the identity of *foxi3a*-positive and *foxi3b-*positive cells, we conducted triple-labeling with a *foxi3a* or *foxi3b* riboprobe together with NaRC- (anti-Na***^+^***,K***^+^***-ATPase) and HRC-specific (anti-H***^+^***-ATPase) antibodies on 72-hpf embryos. The results showed that *foxi3a* was indeed specifically expressed in HRCs (Figure 1L, red arrow), while *foxi3b* was weakly expressed in HRCs (Figure 1M, red arrow) but strongly expressed in NaRCs (Figure 1M, green arrow). This observation demonstrated that *foxi3a* and *foxi3b* are good markers not only for epidermal IC progenitors (before 14 s) but also for respectively differentiating HRCs and NaRCs. By cross-referencing the marker gene expression as well as the apical opening of epidermal ICs (see Figure S1 for details), we categorized three major development events of epidermal ICs in zebrafish as the progenitor stage (from the 90% epiboly to the 14-s stage), differentiation stage (from the 14-s stage to 36 hpf), and maturation stage (from 36 hpf onwards). According to the successive expression of the progenitor markers (*foxi3a* and *foxi3b)* as well as differentiation markers (*atp1b1b* and *ca2a*), we proposed that NaRCs and HRCs may be generated from common progenitors by the differentially regulated expressions of *foxi3a* and *foxi3b* (summarized in Figure 1N).

### Lateral Inhibition Plays a Role in Restricting the Number of Epidermal IC Progenitors

In zebrafish embryos, *foxi3a*-positive epidermal IC progenitors appear on the IC domain as early as the 90% epiboly stage. By examining the expressions of *foxi3a* and epidermal SC markers of P63 [28], we observed that *foxi3a* is co-expressed in a subpopulation of P63-positive cells at the tb stage (Figure 2A, labeled by asterisks). However, from the tb stage onwards, nuclear P63 staining in *foxi3a*-positive IC progenitors was gradually downregulated and eventually reached an undetectable level by 24 hpf (Figure 2B and 2C). This observation suggests that epidermal IC progenitors may be generated from the epidermal SC. We were interested in the molecular mechanism controlling the cell fate choice between epidermal ICs and epidermal SCs. Based on the mosaic pattern of epidermal ICs and epidermal SCs (Figure 2C), we speculated that a D-N-mediated lateral inhibition mechanism may be involved. To test this hypothesis, we initially treated embryos with the *γ*-secretase inhibitor, DAPT, to inhibit Notch proteolysis, nuclear translocation, and signaling [29]. The DAPT-treated or control embryos were then fixed at the tb stage, and the *foxi3a* expression area was quantified by imaging software (see Materials and Methods). By comparing the sporadic pattern in wild-types (Figure 2D) or the DMSO sham control (data not shown), it was noted that *foxi3a* expression in DAPT-treated embryos had become about 2.2-fold denser and more homogeneous (Figure 2E; also summarized in Table 1). This result suggests that Notch signaling might play a role in regulating the IC progenitor number at the onset of IC specification. To confirm this preliminary observation, we examined *foxi3a*-positive epidermal IC progenitors in an antimorphic allele of *mind bomb* mutants (*mib^ta52b^*), which fails in lateral inhibition due to widely compromised Notch signaling and results in premature differentiation of neural progenitors and neuron mass-production [30], [31]. We also detected about 2.3-fold denser *foxi3a* expression in *mib^ta52b^* mutants (Figure 2G; Table 1) than siblings. In addition, in both DAPT-treated and *mib^ta52b^* embryos, loss of Notch activity was confirmed by desynchronized *deltaC* expression in somite stripes (data not shown). Therefore, we confirmed that Notch signaling indeed plays a role in restricting the number of epidermal IC progenitors in zebrafish embryos.

**Table 1:** Quantitative measurement of *foxi3a* or *deltaC* expression in ventral ectoderm of treated embryos aged at tail bud stage

Treatment	*foxi3a*+area (µm^2^)	N	*deltaC*+area (µm^2^)	N
wild types	48490±9499	17	18321±7866	17
GFP RNA	48733±10014	17	NA	
DAPT	105347±11502 ^*^	27	NA	
*mib^ta52b^*	113311±15815 ^*^	34	105268±20847 ^*^	10
*X-Su(H)^DBM^* RNA	99793±17659 ^*^	17	71457±19542 ^*^	13
*X-Su(H)/Ank* RNA	1942±2550 ^*^	17	27±74 ^*^	18
*bea^tit446^*	120642±23994 ^*^	30	100661±28423 ^*^	10
*bea^tw212b^*	113259±8829 ^*^	30	103544±11996 ^*^	10
*des^th35b^*	68249±8898 ^*^	34	68046±20224 ^*^	16
*notch1b* MO	44990±5257	21	NA	
*notch3* MO	44574±6862	21	NA	
*notch1b* MO+*des^th35b^*	63949±10181 ^*^	10	NA	
*notch3* MO+*des^th35b^*	106885±13361^*^	17	NA	
*notch1a* ICD RNA	2174±2750 ^*^	23	2008±1542 ^*^	20
*notch3* ICD RNA	878±1974 ^*^	11	NA	

N, number of embryos scored. NA, not assayed. The values are shown as means±SD. ^*^, significant difference (Student's *t*-test, p<0.05) comparing with wild types.

Whether epidermal ICs behave as D-N signal-sending or -receiving cells was unknown. We attempted to determine this by manipulating the Suppressor of Hairless [Su(H)] activity with mRNAs encoding either the dominant-active form of *X-Su(H)/Ank* or the dominant-negative form of *X-Su(H)^DBM^* [32], The Su(H) is a component downstream of Notch signaling and functions as an effector to prevent receiving cell from adopting a default cell fate. If epidermal ICs behave as D-N signal-sending cells, it is expected that excessive epidermal ICs will be detected in *X-Su(H)^DBM^* mRNA-injected embryos (with reduced Notch activity). On the contrary, if epidermal SCs behave as D-N signal-sending cells, excessive epidermal ICs should appear in *X-Su(H)/Ank* mRNA-injected embryos (with enhanced Notch activity). Our results showed that *foxi3a*-positive epidermal ICs were sharply reduced in or even completely absent from *X-Su(H)/Ank* mRNA-injected embryos (Figure 2I; Table 1), while *foxi3a* expression in *X-Su(H)^DBM^* mRNA-injected embryos became about 2-fold denser (Figure 2H; Table 1). The reduced or excessive epidermal IC phenotype is unlikely to be due to toxicity when *X-Su(H)/Ank*- or *X-Su(H)^DBM^*-mRNA is misexpressed, since no detrimental effect was observed in GFP-misexpressing embryos (Figure 2E; Table 1). Therefore, we concluded that epidermal ICs behave as D-N signal-sending cells while epidermal SCs serve as signal-receiving cells. Interestingly, in DAPT-treated, *mib^ta52b^* or *X-Su(H)^DBM^* mRNA-injected embryos, the *foxi3a*-positive epidermal IC progenitors only appeared within the epidermal IC domain, but never extended outside the domain. This result suggests that there should be other mechanisms which delineate the *foxi3a*-positive epidermal IC domain first, and the D-N-mediated lateral inhibition subsequently functions within this domain to single out epidermal IC progenitors from epidermal SCs.

### The Excessive Epidermal IC Phenotype in *mib^ta52b^* Mutants is at the Expanse of Epidermal SCs

**Figure 3:**
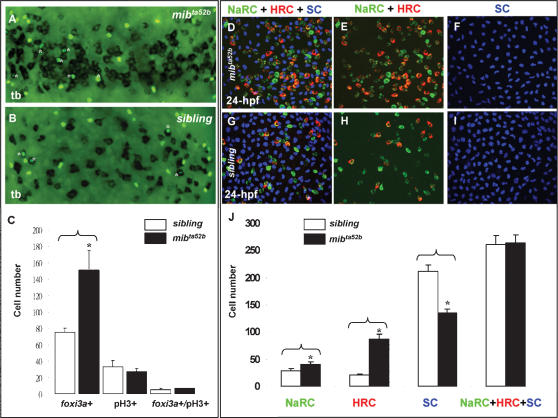
Excess Epidermal Ionocytes Detected in *mib^ta52b^* Occur at the Expanse of Epidermal Stem Cell Fate. (A–C) Detection of the cell proliferation activity within the epidermal ionocyte domain between *mib^ta52b^* and siblings at the tail bud (tb) stage. For *mib^ta52b^* embryos (B) or their siblings (A), epidermal ionocyte progenitors were labeled with *foxi3a* (black), and mitotic cells were labeled with phosphor-histone 3 (pH3) antibody staining (green). Cells which were double positive for *foxi3a* and pH3 are highlighted by asterisks. (C) Quantitative comparison of the epidermal ionocyte progenitor number, mitotic divided cell number, and mitotic divided epidermal ionocytes between *mib^ta52b^* embryos and siblings. (D–I) The excess epidermal ionocytes in *mib^ta52b^* embryos occur at the expense of the epidermal stem cell fate. For 24-hour post-fertilization (hpf) *mib^ta52b^* embryos (D–F) and siblings (G–I), differentiating Na^+^,K^+^-ATPase-rich cells (NaRCs), H^+^-ATPase-rich cells (HRCs), and epidermal stem cells were labeled with *atp1b1b (green), ca2a*, (red), and P63 (blue) staining, respectively. (J) Quantitative comparison of NaRC, HRC, and epidermal stem cell numbers between *mib^ta52b^* embryos and siblings. The cell number in (C and J) is presented as the mean±S.D. *, *p*<0.05, compared with siblings, as determined by Student's *t*-test.

The excessive epidermal IC phenotype observed in either *mib^ta52b^*, DAPT-treated, or *X-Su(H)^DBM^* mRNA-injected embryos might be generated by excessive cell division or by the cell fate choice. We examined cell division of *foxi3a*-stained embryos using phospho-histone 3 (pH3) antibody staining. Within the epidermal IC domain, we counted the number of *foxi3a*-positive epidermal ICs in *mib^ta52b^* mutants and found that it was about 2-fold higher than that of its siblings (151±24 vs. 75±5, *n* = 7, *p*<0.05). However, the proliferation activity in whole embryos (28±4 vs. 33±8, *n* = 7) or in epidermal IC progenitors (7±0 vs. 5±2, *n* = 7) showed no significant difference between *mib^ta52b^* and sibling embryos (Figure 3A–3C). Therefore, it is unlikely that the excessive epidermal IC phenotype observed in *mib^ta52b^* mutants is generated by an excess of cell proliferation. Is it due to an aberrant cell fate choice? By counting the number of epidermal ICs (including both NaRCs and HRCs) and epidermal SCs (by P63 antibody staining) at 24 hpf, we detected significantly higher epidermal ICs (NaRCs, 41±5 vs. 28±5, *n* = 7, *p*<0.05; HRCs, 88±8 vs. 21±3, *n* = 7, *p*<0.05) and lower epidermal SCs (135±8 vs. 212±11, *n* = 7, *p*<0.05) in *mib^ta52b^* mutants. When the total number of epidermal ICs and epidermal SCs was taken into account, there was no significant difference between *mib^ta52b^* and sibling embryos (Figure 3D–3J). These results clearly demonstrate that excessive epidermal ICs occur at the expense of epidermal SC cell fate, and also suggest the default cell fate of the IC domain is set as epidermal ICs when the Notch signal is absent. However, when Notch activity is enhanced, the entire epidermal IC domain will adopt an epidermal SC cell fate. Therefore, we concluded that a balanced mixture of epidermal ICs and epidermal SCs within the IC domain is maintained by the D-N-mediated lateral inhibition mechanism.

### Lateral Inhibition of Singling-out Epidermal IC Progenitors is Mediated by the DeltaC Ligand and Notch1a/Notch3 Receptors

**Figure 4:**
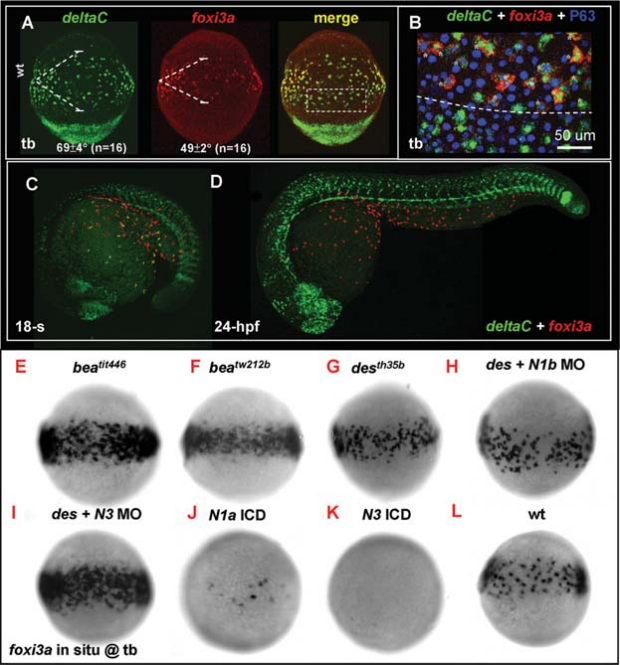
Lateral Speciation on Singling-out Epidermal Ionocyte Progenitors is Mediated by *deltaC* Ligand and *notch1a/notch3* Receptors. (A) Fluorescence double *in situ* hybridization shows the overlapping expression between *deltaC* (green, left) and *foxi3a* (red, middle) on the epidermal ionocyte domain of the ventral ectoderm at the tail bud (tb) stage. The angles of the *deltaC* and *foxi3a* expression domain are presented as the mean±S.D. (B) The area demarcated by the dotted line in (A) is viewed at high magnification. Basically, *foxi3a*+ epidermal ionocyte progenitors (red) also co-express *deltaC* (green, asterisks). However, some *deltaC*+ cells outside the epidermal ionocyte domain are negative for *foxi3a*, suggesting that they are not epidermal ionocytes. (C–D) Fluorescent double *in situ* hybridization with *deltaC* (green) and *foxi3a* (red) probes to show that d*eltaC* is transiently expressed in the epidermal ionocyte lineage. As development proceeds, *deltaC* is sharply downregulated in the epidermal ionocyte lineage. (E–L) Evaluation of *foxi3a* expression by genetic mutants or morphants with reduced or enhanced Notch activity at the tb stage. *foxi3a* expression in the epidermal ionocyte domain was more homogeneous in *deltaC* mutants of *bea^tit446^* (E) and *bea^tw212b^* (F), in a *notch1a* mutant of *des^th35b^* (G), and in a *notch3* MO-injected *des^th35b^* mutant (I). The *foxi3a* expression in the epidermal ionocyte domain was severely reduced in *notch1a* intracellular domain (ICD) RNA- (J) or *notch3* ICD RNA-injected embryos (K). (H) *notch1a*/*des ^th35b^* mutants injected with the *notch1b* MO showed no significant difference with uninjected mutants. N1a, notch1a; N1b, notch1b; N3, notch3; ICD, intra-cellular domain; MO, morpholino.

D-N-mediated lateral inhibition plays essential roles in the cell fate choice and cell differentiation of diverse target tissues by utilizing different combinations of Delta/Jagged ligands and Notch receptors in a stepwise manner [33], [34]. In zebrafish, at least eight *delta/jagged* genes: *deltaA* [35], *deltaB* [36], *deltaC* [37], *deltaD* [38], *delta-like 4 (dll4)* [39], *jagged1a* [40], *jagged1b* [40], and *jaggged2* [36], [40], and four *notch* genes: *notch1a* [41], *notch1b* [42], *notch2* [42], and *notch3*, [42] have been identified so far. To figure out which combination of ligand and receptor is used, we performed *in situ* hybridization with *deltaA, deltaB, deltaC, deltaD, dll4, jagged1a, jagged1b,* and *jaggged2* probes to look for possible *delta/jagged* components expressed on epidermal IC progenitors around the tb to 3-s stages. As a result, only *deltaC* was detected as being expressed on the ventral ectoderm in a salt-and-pepper pattern (Figure S2). This result suggests that *deltaC* may be the only ligand involved in mediating IC specification. In zebrafish, although the epidermal expression of *deltaC* has previously been described [37], the identity of *deltaC*-positive cells is unknown. To validate whether *deltaC* is expressed in the epidermal IC lineage, we performed fluorescent double *in situ* hybridization with *deltaC* and *foxi3a* probes on zebrafish embryos at the tb stage. Results demonstrated that both *foxi3a* and *deltaC* were largely co-expressed in epidermal IC progenitors (Figure 4A). However, we noted that the territory (expressed as the degree of the angle shown in Figure 4A) of *deltaC*-positive cells (69°±4°, *n* = 16) was greater than that of *foxi3a*-positive cells (49°±2°, *n* = 11). A high-magnification view confirmed that most *deltaC*-positive cells outside the IC domain were indeed *foxi3a-*negative (Figure 4B). We speculated that these *deltaC*-positive/*foxi3a*-negative cells might be the progenitors for other epidermal cell types. It was also noted that *deltaC* expression on the epidermal IC lineage was relatively transient compared to that of *foxi3a* (Figure 4C and 4D). By 24 hpf, when *foxi3a* was robustly being expressed, *deltaC* was sharply downregulated to an undetectable level in the epidermal ectoderm (Figure 4D).

Next, we searched for possible Notch receptors by performing *in situ* hybridization with *notch1a, notch1b, notch2,* and *notch3* probes. Results showed that *notch1a* was diffusely and strongly expressed in the ventral ectoderm (Figure S3) and possibly functions as a receptor to receive DeltaC signals. Other *notch* genes, on the contrary, were only expressed in the ventral ectoderm at a basal level (Figure S3). To further validate the right components for transmitting lateral inhibition for selecting epidermal IC progenitors, we assayed the loss-of-function phenotypes of *deltaC, notch1a, notch1b, notch2*, and *notch3* genes in genetic mutants or morphants by examining their *foxi3a* expression at the tb stage. Results confirmed that *deltaC* is indeed a crucial ligand and the only one which sends a lateral inhibition signal to single out epidermal IC progenitors, since the entire IC domain homogeneously expressed *foxi3a* in *deltaC*/*bea^tit446^* (Figure 4E; Table 1) and *deltaC*/*bea^tw212b^* mutants (Figure 4F; Table 1). In *notch1a*/*des^th35b^* mutants, we also detected an increase in *foxi3a* expression within the IC domain (Figure 4G; Table 1). However, its *foxi3a*-positive area was much less dense than those of *mib^ta52b^*, *bea^tit446^*, or *bea^tw212b^* mutants (Table 1). These results suggest that there should be other *notch* receptor(s) which synergistically function with *notch1a* in mediating lateral inhibition. We explored this possibility by injecting *notch1b* morpholino (MO), *notch2* MO, or *notch3* MO into *des^th35b^* mutants and assaying *foxi3a* expression at the tb stage. As a result, we failed to detect any significant increase in the *foxi3a*-positive area when *notch1b* MO (Figure 4H; Table 1) or *notch2* MO (data not shown) was injected into *des^th35b^* mutants. However, when *notch3* MO was injected into *des^th35b^* mutants, the entire IC domain homogeneously expressed *foxi3a* (Figure 4I; Table 1). In addition to the loss-of-function assay, we also validated the functions of *notch1a* and *notch3* through a gain-of-function approach. As we expected, the *foxi3a*-positive area was greatly reduced or even completely lost when we enhanced Notch activity by misexpressing intracellular domains (ICD) of either *notch1a* (Figure 4J; Table 1) or *notch3* (Figure 4K; Table 1). Taken together, we concluded that *notch1a* plays a more-predominant role than *notch3* in receiving *deltaC* signals from epidermal ICs. In addition, the cooperation between *notch1a and notch3* carries out D-N-mediated lateral inhibition in a synergistic manner to maintain a balanced population of epidermal ICs and SCs.

### *deltaC* Expression in Epidermal IC Progenitors is Positively and Negatively Regulated by *ascl1a* and *notch*, Respectively

**Figure 5:**
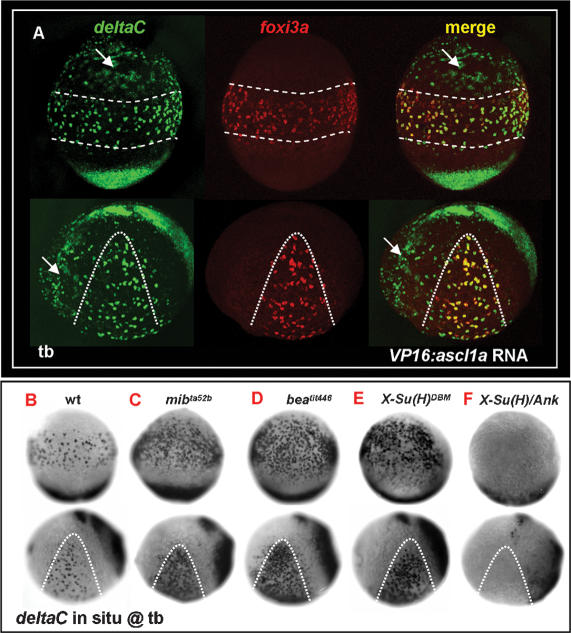
*deltaC* is Positively Regulated by *ascl1a* and Receives Negative Feedback by *notch.* (A) Misexpression of *VP16:ascl1a* mRNA was sufficient to generate ectopic *deltaC* expression outside the epidermal ionocyte domain. *foxi3a* expression, on the contrary, was not affected by *VP16:ascl1a* mRNA misexpression. (B–F) Evaluation of *deltaC* expression by genetic mutants or mRNA-injected embryos with enhanced or reduced Notch activity. Compared to the wild-type (B), *deltaC* expression in the epidermal ionocyte domain was more homogeneous in *mib^ta52b^* (C), *bea^tit446^* (D), and *X-Su(H)^DBM^* mRNA-injected embryos (E). On the contrary, *deltaC* expression in the epidermal ionocyte domain was completely abolished in *X-Su(H)/Ank* mRNA-injected embryos (F). Embryos in the upper panel of all photos are oriented in ventral view, with the anterior to the top, while in the lower panel, all are oriented in lateral view, with the anterior to the left. Epidermal ionocyte domains are highlighted by dotted lines. hpf, hour post-fertilization; tb, tail bud.

In *Drosophila*, the *delta* expression in signal-sending cells is positively enhanced by a proneuronal gene but negatively regulated by Notch activity [18], [19]. This feedback regulatory loop can amplify the tiny difference in Delta expression between cells in a tissue at the beginning of the cell fate choice stage, and gradually specifies the cell identity between pure signal-sending and -receiving cells. In zebrafish, we were curious about whether the initial *deltaC* expression in epidermal IC progenitors is also positively activated by a proneural gene and negatively regulated by the Notch negative regulatory loop. To test this hypothesis, we first forced expression of the *ascl1a* (*achaete-scute complex-like 1a*) proneural gene (also expressed in epidermal IC progenitors, data not shown) by mRNA injection and assayed *deltaC* expression at the tb stage. Results showed that *deltaC* was upregulated by *ascl1a* mRNA misexpression; however, the penetrance was low (data not shown). To exaggerate the function of *ascl1a*, we fused a strong activation domain from a herpes simplex virus with full-length *ascl1a* cDNA to generate *VP16:ascl1a* chimera constructs [43]. Interestingly, when enhanced versions of *VP16:aslc1a* mRNA were forcedly expressed in zebrafish embryos, this was sufficient to induce ectopic *deltaC* expression in the cephalic epidermal ectoderm outside the IC domain (Figure 5A, green panel, indicated by arrows), but insufficient to induce *foxi3a* expression in this ectopic site (Figure 5A, red panel). These results indicated that, first, *ascl1a* can regulate *deltaC* but not *foxi3a* expression; and second, ectopic *deltaC*-expressing cells outside the IC domain are not epidermal ICs.

We also manipulated Notch activity to see whether *deltaC* expression is negatively regulated. Compared to wild-type embryos (Figure 5B), we found that the *deltaC*-positive area in the ventral ectoderm was 5.7-fold increased in *mib^ta52b^* (Figure 5C; Table 1), 5.5-fold increased in *des^tit446^* (Figure 5D; Table 1) and 3.9-fold increased in *X-Su(H)^DBM^* mRNA-injected embryos (Figure 5E; Table 1), whose Notch activity was compromised due to a failure of *deltaC* ubiquitination, *deltaC* ligand expression, and transmission of the Notch signal through Su(H), respectively. On the contrary, in *X-Su(H)/Ank* mRNA-injected embryos whose Notch signal was enhanced, *deltaC* expression in the ventral ectoderm was completely abolished (Figure 5F; Table 1). Therefore, we confirmed that *deltaC* expression by epidermal IC progenitors is positively and negatively regulated by *ascl1a* and *notch*, respectively.

### Reciprocal Regulation of *foxi3a* and *foxi3b* Expressions by a Positive Regulatory Loop

**Figure 6:**
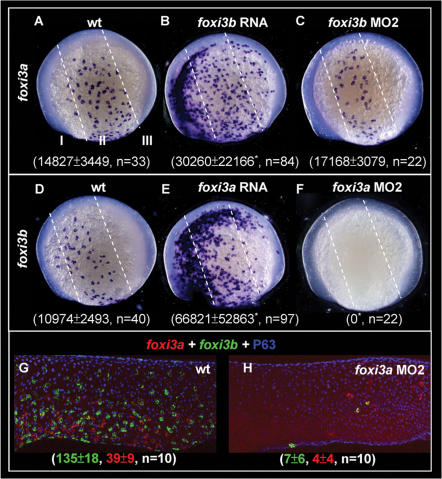
Reciprocal Regulation of *foxi3a* and *foxi3b* by a Positive Feedback Regulatory Loop. (A–C) Detection of *foxi3a* expression in wild-type (wt) embryos, *foxi3b* mRNA-injected embryos, and *foxi3b* morphants at the 5-somite (5-s) stage. (D–F) Detection of *foxi3b* expression in wt embryos, *foxi3a* mRNA-injected embryos, and *foxi3a* morphants at the 5-s stage. The *foxi3a+* or *foxi3b*+ area was measured by ImageJ software and is presented as the mean±S.D (µm^2^). Asterisks indicate *p*<0.05 when compared with the wild-type embryos, as determined by Student's *t*-test. To highlight the position of ectopic epidermal ionocytes which were generated by misexpressing either *foxi3a* or *foxi3b*, the epidermal ectoderm is subdivided into three domains of I, II and III by dotted lines. (G–H) Comparison of *foxi3a* (red) and *foxi3b* (green) expressions between 24-hour post-fertilization (hpf) wt embryos and *foxi3a* morphants. The number of *foxi3a*-(red) and *foxi3b*-(green) expressing ionocytes is indicated in the bottom.

*foxi3a* is activated earlier than *foxi3b* in epidermal IC progenitors (Figure 1N). We tested the hierarchical relationship between *foxi3a* and *foxi3b* by both loss- and gain-of-function approaches in 5-s embryos. Compared to wild types (*foxi3a,* Figure 6A; *foxi3b*, Figure 6D), we found that *foxi3a* expression was almost unaffected in *foxi3b* morphants (Figure 6C), while *foxi3b* expression was completely abolished in *foxi3a* morphants at the 5-s stage (Figure 6F). However, as development proceeded, it was still possible to detect some *foxi3a*- or *foxi3b*-positive epidermal IC progenitors in *foxi3a* morphants, although their relative number was greatly reduced (Figure 6H) when compared to the wild type (Figure 6G). For the gain-of-function assay, *foxi3b* expression was 6.1-fold upregulated in *foxi3a* mRNA-injected embryos (Figure 6E). Surprisingly, *foxi3a* expression was 2-fold upregulated when *foxi3b* mRNA was forcedly expressed (Figure 6B). This result suggests that, first, *foxi3a* is genetically upstream of *foxi3b* and, second, *foxi3a* and *foxi3b* can be reciprocally regulated by a positive feedback loop.

The epidermal ectoderm of zebrafish gastrula can be divided into three subdomains along the A/P axis according to the differential expressions of *gata2* and *gata3* [44]. After completion of convergent extension, these three subdomains give rise to the epidermis covering the cephalic (zone I), trunk (zone II), and tail (zone III), respectively. For wild-type 5-s embryos, we found that *foxi3a* (Figure 6A) and *foxi3b* (Figure 6D) were restrictedly expressed within the epidermal IC domain (corresponding to zone II). However, either *foxi3a* (50%, *n* = 255, Figure 6E) or *foxi3b* (35%, *n* = 223, Figure 6B) misexpression was sufficient to generate extra epidermal IC progenitors at ectopic sites of zones I and III at the 5-s stage. Moreover, the generating potential of ectopic epidermal ICs gradually declined from zones I to III. This result demonstrated that both *foxi3a* and *foxi3b* are sufficient to specify the IC identity and that the entire epidermal ectoderm is permissive for epidermal IC formation.

**Figure 7:**
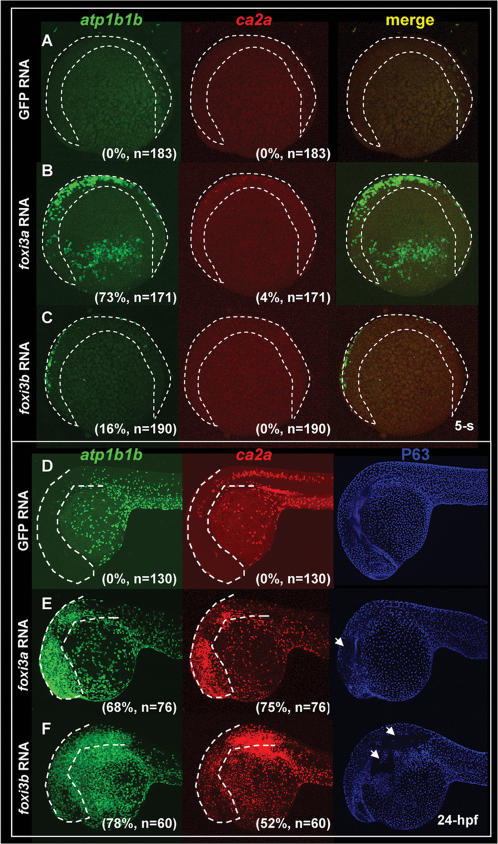
Misexpression of Either *foxi3a* or *foxi3b* in a Wild-type Background is Sufficient to Ectopically Generate Precocious Differentiated Ionocytes in the Epidermis. (A–C) Fluorescent double *in situ* hybridization with *atp1b1b* (green) and *ca2a* (red) probes on wild-type embryos, *foxi3a* mRNA-injected embryos, and *foxi3b* mRNA-injected embryos aged at the 5-somite (5-s) stage. For wild-type embryos, normal Na*^+^*,K*^+^*-ATPase rich cell (NaRC) (*atp1b1b+*) and H*^+^*-ATPase rich cell (HRC) (*ca2a+* and *atp1b1b+*) differentiation was not activated until the 14-s and 18-s stages, respectively. However, when *foxi3a* mRNA was misexpressed in wild-type embryos, it was sufficient to promote the precocious differentiation of both NaRCs and HRCs in the ventral ectoderm and ectopic sites (labeled by a dotted line) at the 5-s stage. For *foxi3b* mRNA-misexpression, it is only sufficient to promote the precocious differentiation of NaRCs in the ventral ectoderm and ectopic sites (label by dotted line) at the 5-s stage. (D–F) Triple labeling of *atp1b1b* (green), *ca2a* (red), and P63 (blue) on wild-type embryos, *foxi3a* mRNA-injected embryos, and *foxi3b* mRNA-injected embryos aged at 24 hours post-fertilization (hpf). For wild-type embryos (D), both NaRCs and HRCs seldom appeared on the cephalic ectoderm (highlighted by a dotted line). However, when either *foxi3a* mRNA (E) or *foxi3b* mRNA (F) was misexpressed in wild-type embryos, it was sufficient to generate the ectopic NaRCs and HRCs on the cephalic ectoderm. Ectopic epidermal ionocytes occur at the expanse of epidermal stem cell fate, since some regions with strong *atp1b1b* and *ca2a* expression completely lacked P63 expression (indicated by arrows).

In addition to functioning as epidermal IC determinants, it is not clear whether the unusually high level of exogenous *foxi3a* or *foxi3b* is related to the precocious differentiation of epidermal IC progenitors. Therefore, we addressed this event by performing fluorescent double *in situ* hybridization with *atp1b1b* and *ca2a* probes in either *foxi3a* mRNA- or *foxi3b* mRNA-injected embryos at the 5-s and 24-hpf stages. In a normal condition, NaRCs and HRCs did not differentiate until the 14- and 18-s stages, respectively. However, when *foxi3a* mRNA was misexpressed, we found that the elevated expression level of *foxi3a* was sufficient to promote precocious differentiation of both NaRCs (73%, *n* = 171) and HRCs (4%, *n* = 171) not only within the epidermal IC domain but also in the ectopic sites of the cephalic epidermis at the 5-s stage (Figure 7B). Only NaRCs differentiated precociously in *foxi3b* mRNA-misexpressed embryos at the 5-s stage (16%, *n* = 190, Figure 7C). But at 24 hpf, ectopic HRCs could be found in the cephalic epidermis in *foxi3b* mRNA-misexpressing embryos (52%, *n* = 60, Figure 7F). In addition, we also noted that ectopic epidermal ICs occurred at the expense of the epidermal SC fate, since the epidermal SC marker of P63 was completely abolished in some regions where high levels of *atp1b1b* and *ca2a* were expressed (Figure 7E and 7F, highlighted by arrows). Taken together, we concluded that *foxi3a* is more potent than *foxi3b* in promoting IC differentiation in wild-type embryos, and that the timing of NaRC and HRC differentiation is strongly dependent on the relative concentrations of *foxi3a* and *foxi3b* accumulated in epidermal IC progenitors.

### Gain-of-Function Assay of *foxi3a* and *foxi3b* in *X-Su(H)/Ank* mRNA-Injected Embryos or *bmp7* Morphants

**Figure 8:**
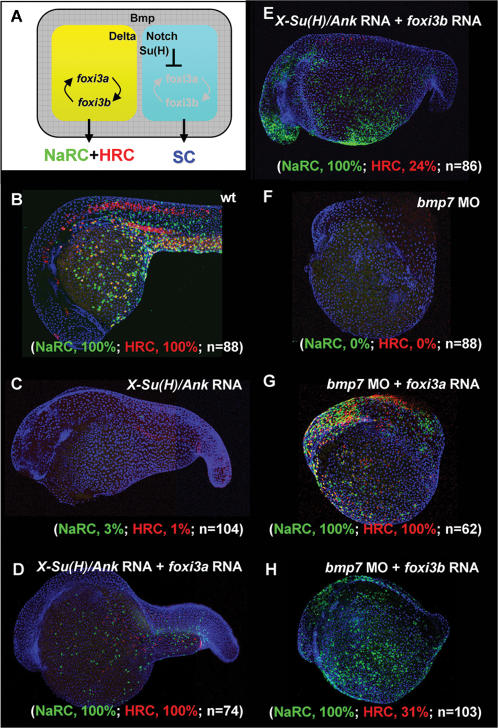
Dissection of the Function of *foxi3a* and *foxi3b* in *X-Su(H)/Ank* mRNA-Injected Embryos or *bmp7* Morphants. (A) Schematic diagram showing the role of Bmp and Delta-Notch signaling in specifying the epidermal ectoderm (gray) and singling-out epidermal ionocytes (yellow) from the epidermal stem cell pool (blue). (B) Normal Na^+^,K^+^-ATPase rich cell (NaRC) and H^+^-ATPase rich cell (HRC) differentiation in wild-type embryos aged at 24 hours post-fertilization (hpf). In wild-type embryos, a few IC progenitors were selected from the epidermal stem cell pool within the epidermal ionocyte domain by Delta-Notch-mediated lateral inhibition, and then differentiated into NaRCs (green, detected by *atp1b1b in situ*) or HRCs (red, detected by *ca2a in situ*) which are scattered on the epidermal layer. (C) For *X-Su(H)/Ank* mRNA-injected embryos (500 pg/embryo), the *foxi3a* and *foxi3b* expressions were inhibited due to the elevated Notch activity in the epidermal ionocyte domain. In such a condition, all epidermal ionocyte progenitors adopted the epidermal stem cell fate; therefore, subsequent NaRC and HRC differentiation was completely abolished. (D) When *foxi3a* mRNA (50 pg/embryo) and *X-Su(H)/Ank* mRNA (500 pg/embryo) were co-injected, high levels of exogenous *foxi3a* expression could compensate for the elevated Notch activity and restore both NaRC and HRC differentiation. (E) When *foxi3b* mRNA (50 pg/embryo) and *X-Su(H)/Ank* mRNA (500 pg/embryo) were co-injected, a high level of exogenous *foxi3b* expression was also sufficient to compensate for the elevated Notch activity to restore NaRC and partially restore HRC differentiation. (F) In *bmp7* morphants (0.1 mM/embryo), although the low level of Bmp signals was still sufficient to promote P63 expression, it was insufficient to drive either *foxi3a* or *foxi3b* expression and finally the epidermal ionocytes lost their identity. (G) When *foxi3a* mRNA (50 pg/embryo) and *bmp7* MO (0.1 mM/embryo) were co-injected, a high level of exogenous *foxi3a* expression could compensate for the low Bmp activity and restore both NaRC and HRC differentiation. (H) When *foxi3b* mRNA (50 pg/embryo) and *bmp7* MO (0.1 mM/embryo) were co-injected, a high level of exogenous *foxi3b* expression could compensate for the low Bmp activity to restore NaRC and partial HRC differentiation. All embryos were scored at 24 hpf.

Based on the differential expressions of *foxi3a* and *foxi3b* in differentiating NaRC (*foxi3b* ^high^) and HRC (*foxi3a* ^high^/*foxi3b* ^low^) lineages, we speculated that they might have distinct functions in promoting the differentiation of NaRCs and HRCs. However, according to the misexpression data performed in wild-type embryos, it was difficult to distinguish the distinct functions between *foxi3a* and *foxi3b* in promoting NaRC and HRC differentiation. Actually, the misexpression results obtained in the wild-type background should be carefully interpreted when both Foxi3a and Foxi3b activities are present, because the positive regulatory loop will amplify downstream *foxi3b* when only *foxi3a* is misexpressed. In the same manner, the positive regulatory loop can also amplify upstream *foxi3a* when only *foxi3b* is misexpressed. Therefore, in the wild-type background, regardless of misexpression *foxi3a* or *foxi3b*, it will turn out to be a combinatorial effect derived from both *foxi3a* and *foxi3b* misexpression. In order to differentiate the functions of *foxi3a* and *foxi3b* more precisely, we sought to create a genetic background which contains no endogenous *foxi3a* or *foxi3b* expression by misexpressing *X-Su(H)/Ank* mRNA or *bmp7* MO. When a high level of *X-Su(H)/Ank* mRNA (500 pg per embryo) was misexpressed, about 45% (*n* = 210) of the injected embryos showed a reduced body axis phenotype, and all the epidermal IC progenitors adopted the epidermal SC fate in such a high Notch activity background (Notch-ON embryos). We selected Notch-ON embryos aged at 24 hpf to perform an *atp1b1b* and *ca2a in situ* study, and found that only 3% of NaRCs and 1% of HRCs (*n* = 104, Figure 8C) differentiated. However, when we forced *foxi3a* expression in Notch-ON embryos, it was sufficient to restore both NaRC (100%, *n* = 74) and HRC (100%, *n* = 74) differentiation with high penetrance (Figure 8D). *foxi3b*, on the contrary, was sufficient to restore full NaRC (100%, *n* = 86) and partial HRC (24%, *n* = 86) differentiation in Notch-ON embryos (Figure 8E).

In parallel, we also dissected the functions of *foxi3a* and *foxi3b* in *bmp7* morphants. Bmp functions as upstream signals, which are essential for ventral cell fate and epidermal ectoderm development [45]. Because epidermal ICs are generated from epidermal SCs, we hypothesized that epidermal IC specification is also regulated by Bmp activity. Indeed, we found that *foxi3a* expression was very sensitive to Bmp activity. In 24-hpf *bmp7* morphants, although we still could detect P63 expression, both NaRC (0%, *n* = 88) and HRC (0%, *n* = 88) differentiations were completely undetectable (Figure 8F) due to the loss of epidermal IC identity (detected by a *foxi3a in situ* study, data not shown). However, when we forced *foxi3a* expression in *bmp7* morphants, it was sufficient to restore both NaRC (100%, *n* = 62) and HRC (100%, *n* = 62) differentiation with high penetrance (Figure 8G). *foxi3b* misexpression was sufficient to restore NaRC (100%, *n* = 103) and partially HRC (21%, *n* = 103) differentiation in *bmp7* morphants (Figure 8H). Taken together, we concluded that first, either Foxi3a or Foxi3b activity may be sufficient to promote the default fate of NaRC differentiation; second, the alternative fate of HRC differentiation requires a high level of Foxi3a; and third, the partial restoration of HRCs in embryos injected with *X-Su(H)/Ank* mRNA+*foxi3b* mRNA or *bmp7* MO+*foxi3b* mRNA may be mediated by Foxi3a activity. And this Foxi3a activity is probably boosted by the Foxi3b feedback regulatory loop.

### Loss-of-Function Assay Suggests that Foxi3a Functions as a Master Regulator to Maintain Epidermal IC Identity and Activate Epidermal IC Differentiation

**Figure 9:**
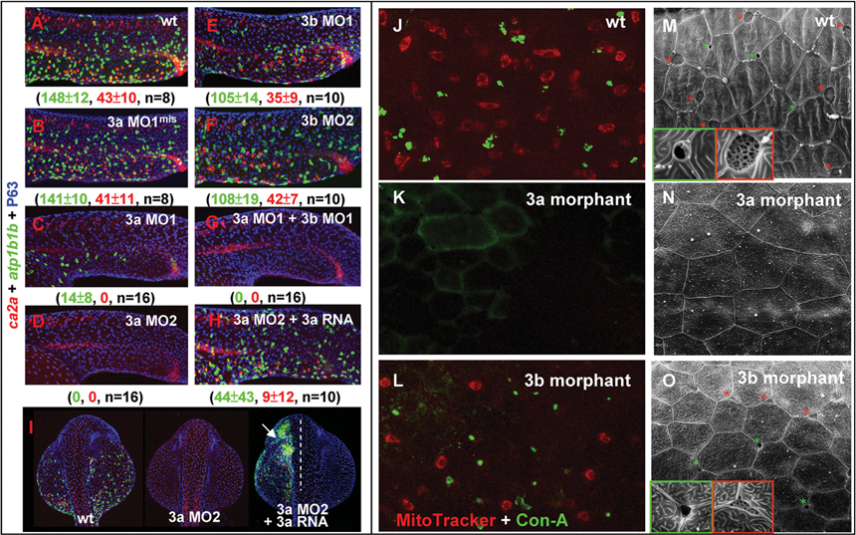
**Knock-down** *foxi3a* Expression Severely Reduces Epidermal Ionocyte Progenitor Number and Abolishes the Later Differentiation Program. (A–G) Interfering with *foxi3a* and *foxi3b* functions of epidermal ionocyte differentiation by a morpholino (0.5 mM/embryo) injection. Morphants were fixed at 24 hours post fertilization (hpf) and stained with *atp1b1b* (green), *ca2a* (red), and P63 (blue) to detect Na*^+^*,K*^+^*-ATPase rich cells (NaRCs), H*^+^*-ATPase rich cells (HRCs), and epidermal stem cells, respectively. (H–I) A rescue experiment to show the specificity of *foxi3a* morpholinos. The *foxi3a* mRNA used for the rescue experiment does not contain a binding site for MO2. (J–L) Comparison of the vital dye uptake ability between the wild type (wt), *foxi3a* morphants, and *foxi3b* morphants. NaRCs and HRCs in either wild-types or *foxi3b* morphants can absorb MitoTracker (red) and Con-A (green) through to their apical openings. For *foxi3a* morphants, no MitoTracker or Con-A staining was detected due to blockage of the entire differentiation program. (M–O) Detection of the apical opening of epidermal ionocytes in wild-types, *foxi3a* morphants, and *foxi3b* morphants by scanning electron microscopy. The apical openings of NaRCs and HRCs in wild-types are shaped as deep holes (green box) or a mesh (red box), respectively. The apical openings were totally undetected in *foxi3a* morphants due to blockage of the entire differentiation program. For *foxi3b* morphants, the apical openings for both NaRCs and HRCs were reduced. Embryos in (A–I) were scored at 24 hpf, while in (J–O), they were scored at 72 hpf. In I, embryos are orientated in a dorsal-up and anterior-top position.

In addition to the gain-of-function assay, we also interfered with *foxi3a* and/or *foxi3b* function by an MO injection. NaRC and HRC differentiation in 24-hpf morphants was then evaluated by fluorescent double *in situ* hybridization with *atp1b1b* and *ca2a* probes. For negative controls, both NaRC and HRC differentiations were largely undisturbed in either *foxi3a* MO^mis^ (Figure 9B) or *foxi3b* MO^mis^ (data not shown). For the weaker *foxi3a* MO1 injection (see Figure S4 for the specificity and efficacy tests), we found that the number of NaRCs was severely reduced to 14±8, while HRCs were completely lost (Figure 9C). With the stronger *foxi3a* MO2 injection, both NaRCs and HRCs were completely abolished (Figure 9D). It is worth noting that the effect of *foxi3a* MO is highly specific to epidermal IC lineages, since the expressional domains of *ca2a* in the pronephric duct and spinal cord neurons, as well as the P63 expression in epidermal SCs were undisturbed in *foxi3a* morphants (Figure 9D). Furthermore, the loss of NaRCs and HRCs in *foxi3a* morphants could be restored by supplying *foxi3a* mRNA (the rescue rate for NaRCs was 95%, and that for HRCs was 56%, *n* = 95; Figure 9H and 9I), indicating that the observed IC phenotype is indeed due to a lack of Foxi3a activity. For *foxi3b* morphants, we found that HRCs were largely undisturbed while NaRCs were slightly reduced when embryos were injected with either *foxi3b* MO1 (Figure 9E), *foxi3b* MO2 (Figure 9F), or a mixture of both *foxi3b* MO1 and *foxi3b* MO2 (data not shown). Why are embryos insensitive to *foxi3b* MO? We propose that the *foxi3a-foxi3b* positive feedback loop can continuously supply *foxi3b* and eventually dilute out the *foxi3b* MO. Interestingly, we found that NaRCs with weaker *foxi3a* MO1 could be completely eliminated when co-injected with the weaker *foxi3b* MO1 (Figure 9G). This result suggests that *foxi3a* and *foxi3b* also function in a synergistic manner to promote epidermal IC differentiation.

There are two possibilities to explain the completely abolished expression of *atp1b1b* and *ca2a* in 24-hpf *foxi3a* morphants: first, epidermal IC identity may have been completely lost; and second, epidermal IC progenitors are still alive but fail to differentiate into NaRCs and HRCs. By examining the progenitor markers of *foxi3a* and *foxi3b*, we learned that epidermal IC identity is reduced but not completely lost in *foxi3a* morphants (Figure 6H). However, surviving epidermal IC progenitors in *foxi3a* morphants are blocked in an undifferentiated status and lose the expression of the differentiating markers of *ndrg1, kcnj1, atp1a1a.2, arg2, trpm7*, and *atp6v0c* (Figure S5). In addition to loss of marker expression, we also provide additional evidence to show that surviving epidermal ICs in *foxi3a* morphants lose their biochemical properties and function. In zebrafish, functionally mature NaRCs and HRCs were able to absorb vital dyes of MitoTracker (Figure 9J, red) and Con-A (Figure 9J, green), respectively, through their unique apical openings [14]. However, we found that 3-dpf *foxi3a* morphants were unable to absorb MitoTracker or Con-A (Figure 9K). On the contrary, *foxi3b* morphants could still mediate MitoTracker or Con-A uptake through mature NaRCs and HRCs (Figure 9L). By a scanning electron microscopic examination, mature NaRCs and HRCs in wild-type embryos were characterized by their distinct types of apical openings in deep-hole (diameter = 2 µm, Figure 9M, green asterisks) or mesh-like shapes (5∼8 µm, Figure 9M, red asterisks), respectively. In *foxi3b* morphants, the apical openings of both NaRCs and HRCs were reduced (Figure 9O), while they were no longer visible in *foxi3a* morphants (Figure 9N).

The specific disappearance of epidermal ICs in *foxi3a* morphants provided an excellent opportunity to evaluate their physiological function *in vivo*. By incubating *foxi3a* morphants in E3 embryonic medium, they suffered severe cardiac and yolk sac edema from 5 dpf onwards (Figure S6C) and eventually died by 10 dpf. If we raised the external osmolarity with isotonic Ringer's buffer, the edema phenotype was greatly reduced (Figure S6D). On the contrary, a more-severe edema phenotype was observed when *foxi3a* morphants were raised in double-distilled water (D2W) (Figure S6E). Therefore, we learned that the edema phenotype in *foxi3a* morphants is primarily caused by unbalanced osmoregulation due to the complete loss of epidermal IC differentiation. By measuring the Ca^2+^ content in morphants, we also provide direct evidence to show that epidermal ICs play an essential role in active Ca^2+^ uptake, since *foxi3a* morphants were unable to maintain Ca^2+^ homeostasis even if high-Ca^2+^ Ringer's solution was provided (Figure S6E). Therefore, our data strengthen the idea that epidermal ICs in fish embryos indeed play a role in maintaining water and ion homeostasis before their branchial counterparts are fully matured.

### Summary of the Epidermal IC Specification and Differentiation Program in Zebrafish

**Figure 10:**
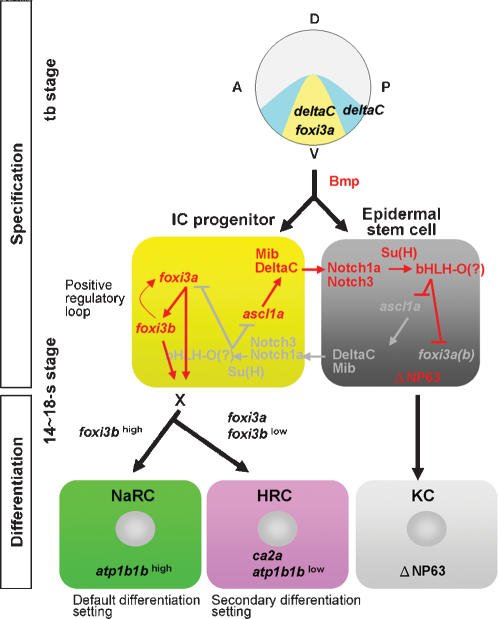
Schematic Diagram Shows the Molecular Mechanism Controlling Epidermal Ionocyte Specification and Differentiation in Zebrafish. For the cell fate specification stage of epidermal ionocyte development, Bmp-DeltaC/Notch1a/3 signals play an essential role in setting the epidermal ectoderm competence and singling-out epidermal ionocyte progenitors from the epidermal stem cell pool. For the epidermal ionocyte differentiation stage, the *foxi3a/foxi3b* regulatory loop activates an unknown downstream factor (X) to promote a later Na*^+^*,K*^+^*-ATPase-rich cell (green) (NaRC) and H*^+^*-ATPase-rich cell (red) (HRC) differentiation program. See the last "Result" section for details of the regulatory mechanism. D, dorsal; V, ventral; A, anterior; P, posterior; The *foxi3a*+/*deltaC*+ epidermal ionocyte domain is labeled in yellow, while the *deltaC* single-positive domain is labeled in light blue. KC, keratinocytes (gray); tb, tail bud.

In this study, we successfully characterized three developmental programs of epidermal ICs in zebrafish as specification (from the 90% epiboly to the 14-s stages), differentiation (from the 14-s stage to 36 hpf), and maturation (from 36 hpf onwards) (summarized in Figure 1). The underlying mechanisms controlling epidermal IC specification and differentiation are summarized in Figure 10. For the specification phase, we found that Bmp signals are essential for epidermal IC formation. Once Bmp activity is reduced, the epidermal IC identity is completely lost, and *foxi3* expression becomes undetectable. In the wild-type, *foxi3a*-positive epidermal IC progenitors are activated on the IC domain of the ventral ectoderm as early as the 90% epiboly stage. D-N-mediated lateral inhibition plays a role in singling-out epidermal IC progenitors, while keeping most cells as epidermal SCs within the IC domain. The epidermal ICs themselves function as signal-sending cells and express high levels of the *deltaC* ligand. The lateral inhibition signal is then transmitted from epidermal ICs to the surrounding epidermal SCs and received by *notch1a* and *notch3* receptors. When *notch1a* and *notch3* are activated in epidermal SCs, the Notch signal is then mediated by Su(H) to activate the unknown member of bHLH-O in inhibiting expression of the pro-differentiating factors of *ascl1a* and *foxi3a(3b)* in the epidermal SC side. The low level of *ascl1a* in epidermal SCs renders *deltaC* unable to transmit the lateral inhibition signal back to epidermal IC progenitors ultimately creating a balanced population of epidermal ICs and epidermal SCs on the ventral ectoderm by such a negative feedback regulatory loop. On the epidermal IC side, upregulation of *foxi3a* activates *foxi3b*, while *foxi3b* also upregulates *foxi3a* expression by a positive feedback loop. By the 10-s stage, the overlapping expressions of *foxi3a* and *foxi3b* in epidermal IC progenitors are separated by unknown factor(s) and they begin to be differentially expressed in different lineages. By gain- and loss-of-function assays, we learned that NaRCs might be set as the primary differentiation fate by *foxi3a*/*foxi3b* function. HRCs, on the contrary, need a higher level of *foxi3a* expression to activate their secondary differentiation fate.

## Discussion

### Epidermal ICs are a New Target Site for D-N Lateral Inhibition

Our studies provide evidence to show that epidermal ICs are a novel target for D-N lateral inhibition, and also highlight a strikingly conserved mechanism of D-N in binary cell fate choice from invertebrates to vertebrates. In all cases, blockage of Notch signaling leads to a failure in lateral inhibition and to a great excess of one cell type at the expanse of another. The supernumerary cell types are neural precursors in the neuronal ectoderm [30], [46], [47], ciliated cells in the *Xenopus* epidermis and zebrafish pronephric duct [48], [49], hair cells in the mouse and zebrafish ear [36], [50], [51], secretory cells in the zebrafish gut [52], luminal cells in the chicken stomach [53], alpha cells in the zebrafish pancreas [34], and ICs in the zebrafish epidermis (this study).

The epidermal layer in aquatic vertebrates is relatively simpler than that of their terrestrial vertebrate counterparts. The epidermal layer in fish embryos, for example, is covered by two layers of cells as the enveloping layer (EVL) and deeper layer (DEL). When development proceeds, the EVL is gradually shed and replaced by a true epidermis from the DEL [54]. Actually, epidermal cells in fish and frog embryos are not as simple as we expected. In addition to epidermal cells, other cells like solitary chemosensory cells [55], mucous cell [56], taste buds [57], ciliated cells [48], and epidermal ICs [14], [58] have also been reported in either fish or frogs. Interestingly, all of them are scattered on the epidermis in a salt-and-pepper pattern. According to the data obtained from studies of epidermal ICs (this study) and ciliated cells [48], such a salt-and-pepper distribution pattern is the outcome of D-N lateral inhibition. Therefore, it will be intriguing to test whether Notch signaling plays a role in the specification and differentiation of solitary chemosensory cells, mucous cells, as well as taste buds in the future.

### The *foxi3a* Expression Pattern on Ventral Ectoderm Supports the Concept of the Ectomere

In vertebrates, the neuromere is patterned by the combination of different signal molecules like *wnt, fgf*, and *bmp* along the A/P axis [59], [60]. Compared to the neural ectoderm, the mechanism of patterning of the epidermal ectoderm has been addressed less fully. Couly and Douarin (1990) transplanted the anterior neural crest and epidermal ectoderm between quail and chicken neurula embryos and found that the presumptive facial and hypobranchial ectoderm is also divided into units along the A/P axis [61]. This observation led them to propose a concept of an “ectomere” to highlight the idea that the epidermal ectoderm is also compartmentalized as its neuromere counterpart in neural anlagen [61]. Afterwards, the prospective epidermal ectoderm in the gastrula stage in both *Xenopus* and zebrafish was identified as being patterned into three subdomains along the A/P axis with different levels of *gata2* and *gata3* expressions [44]. Recently, Matsuo-Takasaki and colleagues (2005) discovered that Xenopus *foxi1a* is expressed in the anterior ventral ectoderm (corresponding to zone I) of the neurula and plays an essential role in ventral specification of the later cephalic ectoderm [62]. In this study, we provide more evidence to strengthen the concept of the ectomere by examining the expression and function of zebrafish *foxi3a*. Endogenous *foxi3a* is activated in the middle ventral ectoderm (corresponding to zone II) during gastrulation and functions to specify epidermal IC identity. However, when exogenous *foxi3a* is pan-embryonically misexpressed, we learned that other zones of the ectomere are also permissive to generating ectopic epidermal ICs but with different potentials. In general, such permissive potential to generate ectopic epidermal ICs gradually declines from the anterior toward the posterior. Currently, the molecular mechanism of the patterning of *foxi3a* expression within the IC domain is unclear. Further studies addressing *fgf, bmp*, and *wnt* signal gradients may provide an answer.

### Foxi3a and Foxi3b Function as Highly Potent Epidermal IC Determinants

Winged helix/forkhead genes encode a subset of the helix-turn-helix class of transcription factors and are involved in a wide range of cell-type specifications and differentiation during organogenesis across diverse animal phyla [20], [21], [22], [23], [24]. In mice, *foxi1* has been reported to function as a master regulator in regulating differentiation of FORE cells in the inner ear [3] and intercalated cells in the urinary system [25]. This observation led Al-Awqati and Schwartz to propose a “kidney-inner ear axis” model to highlight the strikingly conserved mechanism of *foxi1* for kidney and inner ear differentiation [2]. In addition to promoting cell differentiation, *foxi1* has also been reported to be a strong cell fate determinant on shaping and patterning the inner ear in zebrafish [26], [63]. Zebrafish *foxi1* is the earliest marker identified as being expressed in the otic placode. Misexpression of *foxi1* is sufficient to activate the expressions of downstream target genes of *pax8, dlx3b, dlx4b*, and *dlx5a* in several ectopic sites [26]. In this study, we also demonstrated that *foxi3a* is the earliest marker expressed in epidermal IC progenitors. *foxi3a* and *foxi3b* are both sufficient to determine the epidermal IC identity by a novel positive regulatory loop. Even in the condition of low Bmp or high Notch activities which does not favor epidermal IC formation, overexpression of either *foxi3a* or *foxi3b* is still sufficient to rebuild the epidermal IC identity as well as to promote a later differentiation program. Why do *foxi* genes function so potently? A recent study revealed that *foxi1* is capable of remodeling chromatin's higher-order structure and can stably create site-specific changes in chromatin to either create or remove DNase I hypersensitive sites [64]. This creates a stable transcriptional “ground state” for cells to rapidly and appropriately respond to external developmental cues. Further studies on the structure and function of Foxi3a by domain analysis will benefit our understanding of its transactivating function.

### Do Foxi3a and Foxi3b Play Distinct Functions in Promoting NaRC and HRC Differentiations?

According to the differential expressions of *foxi3a* and *foxi3b* in differentiating epidermal ICs, *foxi3a* and *foxi3b* may play different roles in HRC and NaRC differentiation. Although the loss-of-function assay suggested that only *foxi3a* but not *foxi3b* is required for NaRC and HRC differentiation, the gain-of-function assay showed that both exogenous *foxi3a* and *foxi3b* are sufficient to promote ectopic NaRC and HRC differentiation in a wild-type background. The results obtained from the gain- and loss-of-function studies do not totally contradict each other when taking the positive feedback regulatory loop between *foxi3a* and *foxi3b* into account. This novel regulatory loop can amplify the downstream *foxi3b* concentration when upstream *foxi3a* is misexpressed, and vice versa. Therefore, regardless of misexpression of *foxi3a* or *foxi3b* in wild-type individuals, the final readout will be the combinatorial results derived from both *foxi3a* and *foxi3b*. By blocking the upstream expression of *foxi3a* in either *foxi3a* morphants, *X-Su(H)/Ank* mRNA-injected embryos, or *bmp7* morphants, the *foxi3a-foxi3b* positive regulatory loop will break down and the entire epidermal IC differentiation program will be abolished. However, when exogenous *foxi3b* is supplied, it is sufficient to restore the primary setting of NaRC differentiation. The secondary setting of HRCs, on the contrary, requires a higher concentration of *foxi3a* for its restoration. This result clearly demonstrates that both *foxi3a* and *foxi3b* are sufficient to promote the primary setting of NaRC differentiation, while the secondary setting of HRC differentiation strongly relies on a higher concentration of *foxi3a*. What is the molecular mechanism which breaks the balanced expression between *foxi3a* and *foxi3b* in common IC progenitors and then drives the lineage separation between NaRCs and HRCs? Our current data are insufficient to answer this basic question. In *Drosophila* SOP development, four daughter cells, socket, hair, sheath and neuronal cells, can be generated from a single SOP by a stepwise asymmetric cell division which is mediated by Notch and Numb interactions [15], [65]. In zebrafish, by systematically analyzing the D-N components, we concluded that *deltaC-notch1a/3*-mediated lateral inhibition plays a role in singling out epidermal IC progenitors from the epidermal SC pool at around the 90% epiboly to tb stages. However, D-N-mediated lateral inhibition might also play a role in later NaRC and HRC lineage separation, since increases in NaRCs and HRCs are not proportional in *mib^ta52b^* but are skewed toward the HRC lineage (Figure 3J). Further studies of NaRC and HRC differentiation on other D-N mutants or morphants will provide insights into this possibility.

In addition, we also noted that *foxi3a* expression is earlier and stronger than that of *foxi3b* in epidermal IC progenitors. If HRC differentiation really relies on a higher concentration of *foxi3a*, then, why don't epidermal IC progenitors differentiate into HRCs as the default differentiation setting? The most reasonable interpretation is that the HRC differentiation program is not directly executed by *foxi3a*. We suggest that *foxi3a* might activate other downstream gene(s) to initiate the HRC differentiation program later than that for NaRCs. Indeed, forkhead genes often collaborate with other transcription factors to control the timing of differentiation, exerting both positive and negative effects on these processes [2]. Therefore, searching for genes which are downstream of or associated with *foxi3* genes by *in situ* screening, mutant screening, microarrays, or a yeast-two-hybrid analysis may provide insights into this topic in the future.

### Epidermal ICs Provide a Simple Model to Study Branchial IC Differentiation

The gills are a high energy-demanding multiple-function organ which contains at least pavement cells (equivalent to keratinocytes), neuroepithelial cells, mucous cells, and branchial ionocytes to mediate the diverse functions of air exchange, oxygen sensing, protection, acid-base balance, and ion transport [5]. Branchial ionocytes in most fish species are concentrated in the interlamellar regions. However, when fish were challenged with lower temperature (MY Chou et al., unpublished observation), hypoxia [66], lower ionic water [67], [68] or a high concentration of a heavy metal [69], branchial ionocytes extend to the more-distal region of the lamella in a short time. However, due to constraints of the complex organization of the gill epithelium and a lack of convincing gene markers to label undifferentiated ionocyte progenitors, whether these ectopic branchial ionocytes are generated from cell migration, cell proliferation, or *in situ* differentiation still remains unknown. In this study, we provide direct evidence to show that *foxi3a* and *foxi3b* are novel markers for epidermal ionocyte progenitors and play essential roles in specifying epidermal IC identity and promoting epidermal IC differentiation in a concentration-dependent manner. This observation raises the possibility that the renewal and differentiation of branchial ionocytes in adult gills might be also mediated by a *foxi3*-dependent pathway. Preliminary data suggest that this answer might be true since we found that *foxi3a* expression and the number of branchial ICs in the secondary lamella were strongly positively correlated (MY Chou et al., unpublished observation). This intriguing finding suggests that ectopic branchial ICs in the secondary lamella might be differentiated from preexisting progenitors. Further studies by manipulating *foxi3a* expression levels in zebrafish gill using transgenic approaches may provide more-direct evidence to support this working hypothesis.

What was the evolutionary driving force to evolve such an elegant, positive regulatory loop between *foxi3a* and *foxi3b* to regulate epidermal IC differentiation? We showed in zebrafish that although the epidermal IC progenitors appear very early at the gastrula stage, they are not functionally mature until 36 hpf based on a Ca^2+^ ion-flux assay [58], an electro-physiological assay [14], vital dye staining, and apical opening observations (this study). Actually, the maturation of epidermal ICs is a very complicated process that involves sequential activation of many genes involved in cell differentiation, ion transport, acid-base regulation, as well as cell shape remodeling [4]. We suggest that the coupled reciprocal regulation between *foxi3a and foxi3b* has at least two benefits for zebrafish embryos, as follows: first, it promotes epidermal IC progenitors entering the differentiation program by rapidly producing high amounts of *foxi3a* and *foxi3b* in a short time frame, and secondarily prevents the complete breakdown of epidermal IC differentiation when the master regulator of *foxi3* function is unilaterally compromised. Therefore, the two duplicate copies of *foxi3* genes may have been preserved during vertebrate evolution by a sub-functionalization mechanism.

## Materials and Methods

### Animals

The wild-type AB strain and mutant lines of zebrafish (*Danio rerio*) were maintained as described previously [70]. Homozygous mutants were obtained by in-crossing between heterozygous carriers for the *mib^ta52b^*, *des^th35b^*, *bea^tit446^*, and *bea^tw212b^* alleles. All animal procedures were approved by the Animal Use and Care Committee of Academia Sinica and A-Star.

### Whole-Mount *in Situ* Hybridization

For single-color *in situ* hybridization, digoxigenin-labeled antisense riboprobes for *foxi3a* [27], *foxi3b* [27], *deltaA* [35], *deltaB* [36], *deltaC* [37], *deltaD* [38], *dll4* [39], *jagged1a* [40], *jagged1b* [40], *jagged2* [36], [40], *notch1a* [41], *notch1b* [42], *notch2* [42], *notch3* [42], *ndrg1* [71], *kcnj1* [72], *atp1a1a.2* [73], *arg2* [74], *trpm7* [75], and *atp6v0c* [76] were generated by *in vitro* transcription, and whole-mount *in situ* hybridization was performed as previously described [77]. Images were taken with a DIC microscope (BX51, Olympus) equipped with a digital camera (DP70, Olympus). For fluorescenct double *in situ* hybridization, *foxi3a* and *ca2a* [72] riboprobes were labeled with digoxigenin, while *foxi3b, deltaC,* and *atp1b1b* [73] riboprobes were labeled with dinitrophenol. We followed the protocol originally described in [78] and improved by [49]. Images were taken with a confocal laser scanning microscope (Fluoview FV1000, Olympus). Since epidermal ICs are located on the superficial epidermis, we found that it was unnecessary to perform proteinase K digestion. To optimize the clarity of the presented panels, the anterior is to the left and dorsal is up in all panels unless otherwise indicated, and scale bars are provided only in the absence of standard anatomic landmarks.

### Whole-Mount Antibody Staining

Whole-mount antibody staining was performed as described previously [14]. Antibodies and their dilutions were used as follows: monoclonal anti-chicken Na***^+^***,K***^+^***-ATPase, 1: 200 (clone α5, Developmental Studies Hybridoma Bank); polyclonal anti-killifish H***^+^***-ATPase, 1: 200 [79]; polyclonal anti-phospho-histone 3 (Ser10), 1: 200 (Santa Cruz); monoclonal anti-human P63, 1: 200 (Santa Cruz); goat anti-mouse IgG-Alexa Flour 488, 1: 200; goat anti-rabbit IgG-Alexa Flour 488, 1: 200; goat anti-rabbit IgG-Alexa Flour 568, 1: 200; and goat anti-mouse IgG-Alexa Flour 647, 1: 200.

### Vital Dye Staining

MitoTracker and Con-A have been utilized as vital dyes to label mitochondria in NaRCs and glycoproteins in the apical openings of HRCs in zebrafish embryos [14]. To evaluate the viability of NaRCs and HRCs in wild-type and morphants, we incubated 3-dpf embryos in 50 ppm of MitoTracker Orange CM-H2TMRos (Molecular Probes, Eugene, OR) or Alexa Fluor 488-conjugated Con-A (Molecular Probes) for 10 minutes in the dark. After extensive washing with E3 embryonic medium (5 mM NaCl, 0.17 mM KCl, 0.33 mM CaCl2, 0.33 mM MgSO4, and 0.1% methylene blue), the stained embryos were observed under a fluorescence microscope (Axioplan2, Zeiss, Germany). The maximum excitation and emission wavelengths for MitoTracker Orange CM-H2TMRos are 554 and 576 nm, and for Alexa Fluor 488-conjugated Con-A are 495 and 519 nm, respectively.

### DAPT Treatment

DAPT treatments were performed as previously described [76]. DAPT (Calbiochem) was reconstituted with dimethyl sulfoxide (DMSO) to make a stock concentration of 100 mM. Aliquots were diluted to 100 µM in E3 embryonic medium. Embryos were placed in the DAPT solution after the 1-K-cell stage and incubated until fixation at the tb stage.

### Plasmid Construction

To generate pCS2+*foxi3a and* pCS2+*foxi3b* constructs, the corresponding *foxi3a* and *foxi3b* coding regions were PCR-amplified with the following pairs of primers: for *foxi3a*, 5′-CGGAATTCATGACATCATTTGTTCCACAG-3′ and 5′-GCTCTAGATTACACCTCAGATCCCTCCCGTGG-3′; and for *foxi3b*, 5′-CGGAATTCATGACATCCTACGAGTCTCAAGG-3′ and 5′-GCTCTAGACTACACCTCTGTGCCTTCCCGAGGG-3′, and cloned into a pCS2+vector [80] at the EcoRI and XbaI sites. To generate the pCS2+*foxi3aUTR:GFP* and pCS2+*foxi3bUTR:GFP* constructs, the corresponding *foxi3a* and *foxi3b* coding regions were PCR-amplified with the following pairs of primers: for *foxi3a*, 5′-CGGAATTCAGCAGAGCAGGAGGCATTTTC -3′ and 5′-GCTCTAGACCCTGCGTATTCTCCGAAATC-3′; and for *foxi3b*, 5′-CGGAATTCGAAGGTCATCAGAGGACAGGAGA-3′ and 5′-GCTCTAGAAGACGGGTTGGTTCTTTGTG-3′, and cloned into a pCS2+GFP XLT vector at the EcoRI and XbaI sites. The final pCS2+foxi3aUTR:GFP and pCS2+foxi3bUTR:GFP vectors containing the 5′UTR and partial exon 1 were in-frame-fused with the GFP reporter. To generate the pCS2+VP16:*ascl1a* construct, the corresponding *ascl1a* coding regions were PCR-amplified with the following pairs of primers: 5′-CGGAATTCAATGGACATCACCGCCAAGATGG -3′ and 5′-GCTCTAGATCAAAACCAGTTGGTGAAGTCC -3′, and cloned into a pCS2+NLS VP16AD vector at the EcoRI and XbaI sites. The corresponding EcoRI and Xba1 sites are underlined.

### mRNA Injection

All constructs cloned in the pCS2+and pCS2+GFP XLT vectors were linearized by Not1, and the capped RNA was transcribed using an SP6 message RNA polymerase kit (Ambion). The pCS2+VP16:*ascl1a* construct was linearized by SacII, and the capped RNA was transcribed by SP6 RNA polymerase. We injected capped mRNA into yolks at the 1∼2-cell stage at the following concentrations: *foxi3a*, 50 pg/embryo; *foxi3b*, 50 pg/embryo; *foxi3aUTR:GFP*, 250 pg/embryo; *foxi3bUTR:GFP*, 250 pg/embryo; *notch1a* ICD [81], 125 pg/embryo; *notch3* ICD [31], 125 pg/embryo; *X-Su(H)/Ank* [32], 500 pg/embryo; *X-Su(H)^DBM^* [32], 500 pg/embryo; and *VP16:ascl1a* [43], 125 pg/embryo.

### Morpholino (MO) Injection and Control

To archive the maximal knock-down effect, 1 nl of serially-diluted MOs (purchased from Gene Tools) at the concentrations of 1, 0.5, 0.25 and 0.1 mM were injected into yolks at the 1∼2-cell stage. The maximal dosage that caused no obvious toxic effect on embryogenesis was used as follows: *foxi3a* MO1 (+72 to+96, against ATG), 5′-AGACTGTGGAACAAATGATGTCATG-3′ at 0.5 mM/embryo; *foxi3a* MO2 (+44 to+68, against 5′UTR), 5′-TCTTCCCGTTTCTCTTTGTTGAAGG-3′ at 0.5 mM/embryo; *foxi3a* MO1^mis ^(+72 to+96, against ATG): 5′-AGAGTGTGCAAGAAATCATGTGATG-3′ at 0.5 mM/embryo; *foxi3b* MO1 (+89 to+113, against ATG), 5′-CCTTGAGACTCGTAGGATGTCATTG-3′ at 0.5 mM/embryo; *foxi3b* MO2 (+64 to+88, against 5′UTR), 5′-CTCGATCCTGAGGGTGCTCCAGTTG-3′ at 0.5 mM/embryo; *foxi3b* MO1^mis^ (+89 to+113, against ATG): 5′-CCTTCACACTCCTAGCATGTGATTG-3′ at 0.5 mM/embryo; *notch1b* MO: 5′-CTCTCCCCATTCATTCTGGTTGTCG-3′ at 0.5 mM/embryo [82]; *notch2* MO: 5′-AGGTGAACACTTACTTCATGCCAAA-3′ at 0.5 mM/embryo [82]; *notch3* MO: 5′-ATATCCAAAGGCTGTAATTCCCCAT-3′ at 0.5 mM/embryo [82]; and *bmp7* MO: 5′-CCAATCCAGAGCAACATCCAGCATG-3′ at 0.1 mM/embryo [83]. To validate the specificity and efficacy of the MOs, we designed two independent MOs against either the translational start site (MO1) or the 5′ untranslated region (UTR) (MO2) and co-injected them with UTR:GFP mRNA into embryos as fluorescent reporters. The UTR:GFP constructs containing the 5′UTR, partial exon 1 of *foxi3a* (or *foxi3b*) was then in-frame-fused with the GFP reporter. Therefore, MO1 and MO2 were expected to simultaneously inhibit the expression of both endogenous *foxi3a* (*foxi3b*) and exogenous GFP in injected embryos. Results showed that both MO1 and MO2 reduced the expression of exogenous *foxi3a (foxi3b)* UTR:GFP, suggesting that they are also able to knock-down the expression of endogenous *foxi3a* or *foxi3b.* The five mismatched control MO1 or MO2 were unable to target exogenous *foxi3a (foxi3b)* UTR:GFP, suggesting that the mis-targeting problem can be ignored. The efficacy of MO2 was superior to that of MO1 at inhibiting the translation of *foxi3a* or *foxi3b* (Figure S4).

### Scanning Electron Microscopy (SEM)

Embryos were pre-fixed with 4% paraformaldehyde in PBS at 4°C overnight and then post-fixed with 2.5% glutaraldehyde and 0.1 M sodium cacodylate again overnight at 4°C under gentle agitation. After rinsing with 0.1 M sodium cacodylate and fixation using 0.1% osmium tetroxide, samples were washed again in 0.1 M sodium cacodylate and then dehydrated stepwise by successive immersion in 50%, 70%, 80%, 90%, 95%, and 100% ethanol. Traces of ethanol in the samples were evaporated with a critical point dryer (HCP-2, Hitachi) and then coated with gold–palladium particles using a sputter coater (Cressington 108). The surface structure of the apical openings was evaluated using an environmental scanning electron microscope (ESEM, FEI Quanta 200).

### Physiology Assay

Initially, we raised morphants and wild-types in E3 embryonic medium from days 0 to 3. On day 3, 100 morphants and wild-type embryos were selected and transferred to Ringer's solution (116 mM NaCl, 2.9 mM KCl, 1.8 mM CaCl2, and 5 mM Hepes; pH 7.2), E3 or double-distilled water in three to five duplications (procedures are highlighted in Figure S6A). On day 7, we scored the survival rate ([number surviving at 7 dpf/normally developing embryos at 3 dpf]×100%) and edema rate ([edematous embryos at 7 dpf/number surviving at 7 dpf]×100%). To measure the whole-body Ca^2+^ content, 30 morphants or wild-type embryos at 7 dpf were selected from double-distilled water-, E3-, and Ringer's solution-acclimated groups and then dehydrated at 65°C overnight. The dry embryos were digested with 1000 µL 13 N HNO_3_ overnight and then subjected to atomic absorption spectrophotometry (Z-8000, Hitachi) following our previously described protocol [58].

### Ionocyte Quantitation and Statistical Analyses

The embryos probed with *foxi3a, foxi3b,* or *deltaC* were oriented in a ventral-up position for the photographs. The gene expression area was quantified using ImageJ software (NIH). Values are presented as the mean (SD) and were compared using Student's *t*-test.

## Supporting Information

Figure S1 Detection of the apical opening of epidermal ionocytes in zebrafish embryos.(A-F) The epidermal layer covering the yolk ball of wild-type embryos was scanned by a scanning electron microscope at different developmental stages (indicated in the lower left-hand corner). The first apical opening of the epidermal ionocyte appeared at 36 hours post-fertilization (hpf) (asterisks).
(8.30 MB TIF)

Figure S2 Screening possible *delta/jagged* ligand expression in the epidermal ionocyte domain. Embryos aged at the tail bud (tb) to 3-somite (3-s) stages were evaluated in situ with either (A) *deltaA*, (B) *deltaB*, (C) *deltaC*, (D) *deltaD*, (E) *delta-like 4*, (F) *jagged1a*, (G) *jagged1b*, or (H) *jagged2* probes. Among the eight *delta/jagged* genes tested, only *deltaC* was detected as being expressed on the epidermal ionocyte domain (highlighted by dotted lines) of the ventral ectoderm.
(9.84 MB TIF)

Figure S3 Screening for possible *notch* receptor expression in the epidermal ionocyte domain. Embryos aged at the tail bud (tb) to the 3-somite (3-s) stages were evaluated *in situ* with either (A) *notch1a*, (B) *notch1b*, (C) *notch2*, or (D) *notch3* probes. Results show that *notch1a* was strongly and ubiquitously expressed in the epidermal ionocyte domain. Other notch genes, on the contrary, were expressed in the ventral ectoderm at a low level.
(6.65 MB TIF)

Figure S4 Control experiments to validate the specificity and efficacy of the morpholino. Schematic diagrams show the relative positions of the designed MOs and reporter constructs for either *foxi3a* (A) or *foxi3b* (B). The 5′UTR and partial exon 1 sequences of *foxi3a* or *foxi3b* were PCR-amplified from zebrafish cDNA and in-frame-fused with the green fluorescent protein (GFP) reporter gene. The resulting chimeric vectors of pCS2+3aUTR:GFP or pCS2+3bUTR:GFP contained the complementary sequences for testing the specificity and efficacy of both MO1 and MO2. (C) When five mismatched controls of 3a MO1^mis^ (0.5 mM) were co-injected with 3aUTR:GFP mRNA (250 pg), all embryos (100%, n = 94) showed strong GFP expression. This result suggests that the 3a MO1mis is unable to target endogenous *foxi3a* mRNA. (D) When the 3a MO1 (0.5 mM) was co-injected with 3aUTR:GFP mRNA (250 pg), GFP expression was completely abolished in 78% of the injected embryos (n = 83). (E) When 3a MO2 (0.5 mM) was co-injected with 3aUTR:GFP mRNA (250 pg), GFP expression was completely abolished in all injected embryos (n = 119). This result suggests that both 3a MO1 and 3a MO2 can target endogenous *foxi3a* mRNA. However, the efficacy of 3a MO2 was superior to 3a MO1. (F) When five mismatched controls of 3b MO1^mis^ (0.5 mM) were co-injected with 3bUTR:GFP mRNA (250 pg), all embryos (100%, n = 94) showed strong GFP expression. This result suggests that the 3b MO1^mis^ is unable to target endogenous *foxi3b* mRNA. (G) When the 3b MO1 (0.5 mM) was co-injected with 3bUTR:GFP mRNA (250 pg), it was only sufficient to abolish GFP expression in 58% of injected embryos (n = 71). (E) When the 3b MO2 (0.5 mM) was co-injected with 3bUTR:GFP mRNA (250 pg), it was sufficient to abolish GFP expression in all injected embryos (n = 145). This result suggests that both 3b MO1 and 3b MO2 can target endogenous *foxi3b* mRNA. However, the efficacy of 3b MO2 was superior to 3b MO1. The number and percentage on the right bottom corner of C-H refer to the ratios of embryos that express GFP. ORF, open reading frame; UTR, untranslated region.
(8.76 MB TIF)

Figure S5 Global down-regulation of epidermal ionocyte markers in *foxi3a* morphants. (A-F) Comparison of the epidermal ionocyte marker expression between wild-type (left panel) and *foxi3a* morphants (right panel). The expression of markers is completely abolished in the epidermal ionocyte lineage in *foxi3a* morphants. Note that the pronephric duct expressions in *ndrg1, kcnj1, atp1a1a.2* and *trpm7* were largely undisturbed, which shows that the *foxi3a* morphant phenotype specifically targets epidermal ionocytes. Na+,K+-ATPase-rich cell (NaRC) markers were *ndrg1* (A), *kcnj1* (B), and *atp1a1a.2* (C). H+-ATPase-rich cell (HRC) markers were *arg2* (D), *trpm7* (E), and *atp6voC* (F). All embryos were scored at 24 hours post-fertilization (hpf).
(9.54 MB TIF)

Figure S6 Role of epidermal ionocytes in water and ion homeostasis in zebrafish embryos. (A) Procedure to assay the physiological functions of epidermal ionocytes in zebrafish embryos. After injecting them with *foxi3a* MO, morphants were initially raised in E3 up to 3 days post-fertilization (dpf) and then challenged with either Ringer's solution, E3, or double-distilled water (D2W). The survival rate, edema rate, and whole-body Ca^2+^ content between wild-types (wt) and morphants were measured at 7 dpf, and results are summarized in (E). The wild-types had a strong water balance ability and showed no edema phenotype in either E3 (B), Ringer's solution, or D2W (not shown). The morphants displayed a severely edematous phenotype in hypotonic E3 (C) or D2W (not shown), while the abnormality was greatly rescued in isotonic Ringer's solution (D). The values are shown as the mean±SD (n = 10). Asterisks (*) indicate a significant difference from the wild-type (Student's *t*-test, *p*<0.05).
(7.09 MB TIF)
