# Supplementary figures and images for: A Positive Regulatory Loop between foxi3a and foxi3b Is Essential for Specification and Differentiation of Zebrafish Epidermal Ionocytes
Source: PLoS One. 2007 Mar 21;2(3):e302. doi: 10.1371/journal.pone.0000302 (PMC1810426; doi:10.1371/journal.pone.0000302)

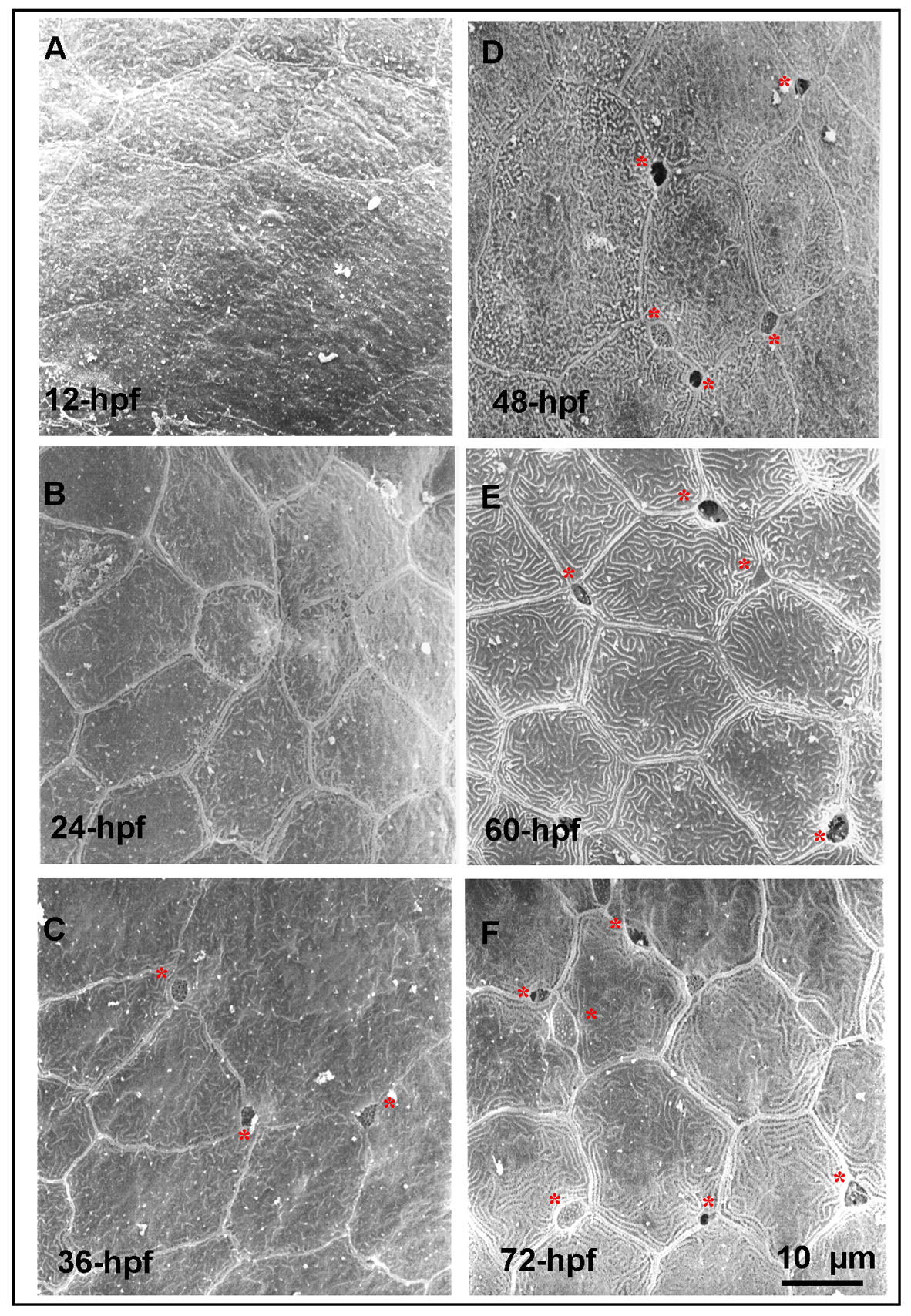

Supplement: Figure S1 — Detection of the apical opening of epidermal ionocytes in zebrafish embryos.(A-F) The epidermal layer covering the yolk ball of wild-type embryos was scanned by a scanning electron microscope at different developmental stages (indicated in the lower left-hand corner). The first apical opening of the epidermal ionocyte appeared at 36 hours post-fertilization (hpf) (asterisks). (8.30 MB TIF) [file pone.0000302.s001.tif]

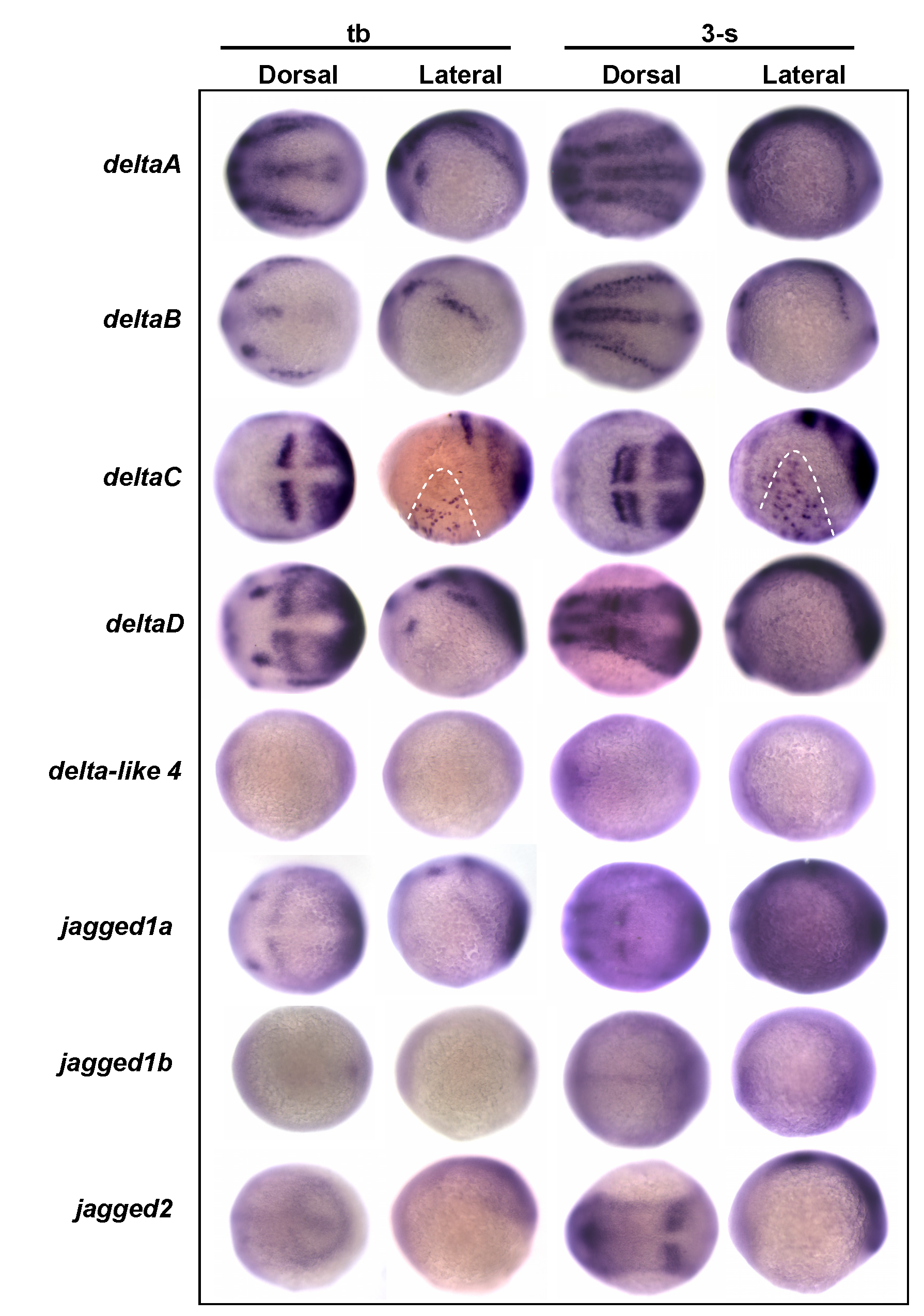

Supplement: Figure S2 — Screening possible delta/jagged ligand expression in the epidermal ionocyte domain. Embryos aged at the tail bud (tb) to 3-somite (3-s) stages were evaluated in situ with either (A) deltaA, (B) deltaB, (C) deltaC, (D) deltaD, (E) delta-like 4, (F) jagged1a, (G) jagged1b, or (H) jagged2 probes. Among the eight delta/jagged genes tested, only deltaC was detected as being expressed on the epidermal ionocyte domain (highlighted by dotted lines) of the ventral ectoderm. (9.84 MB TIF) [file pone.0000302.s002.tif]

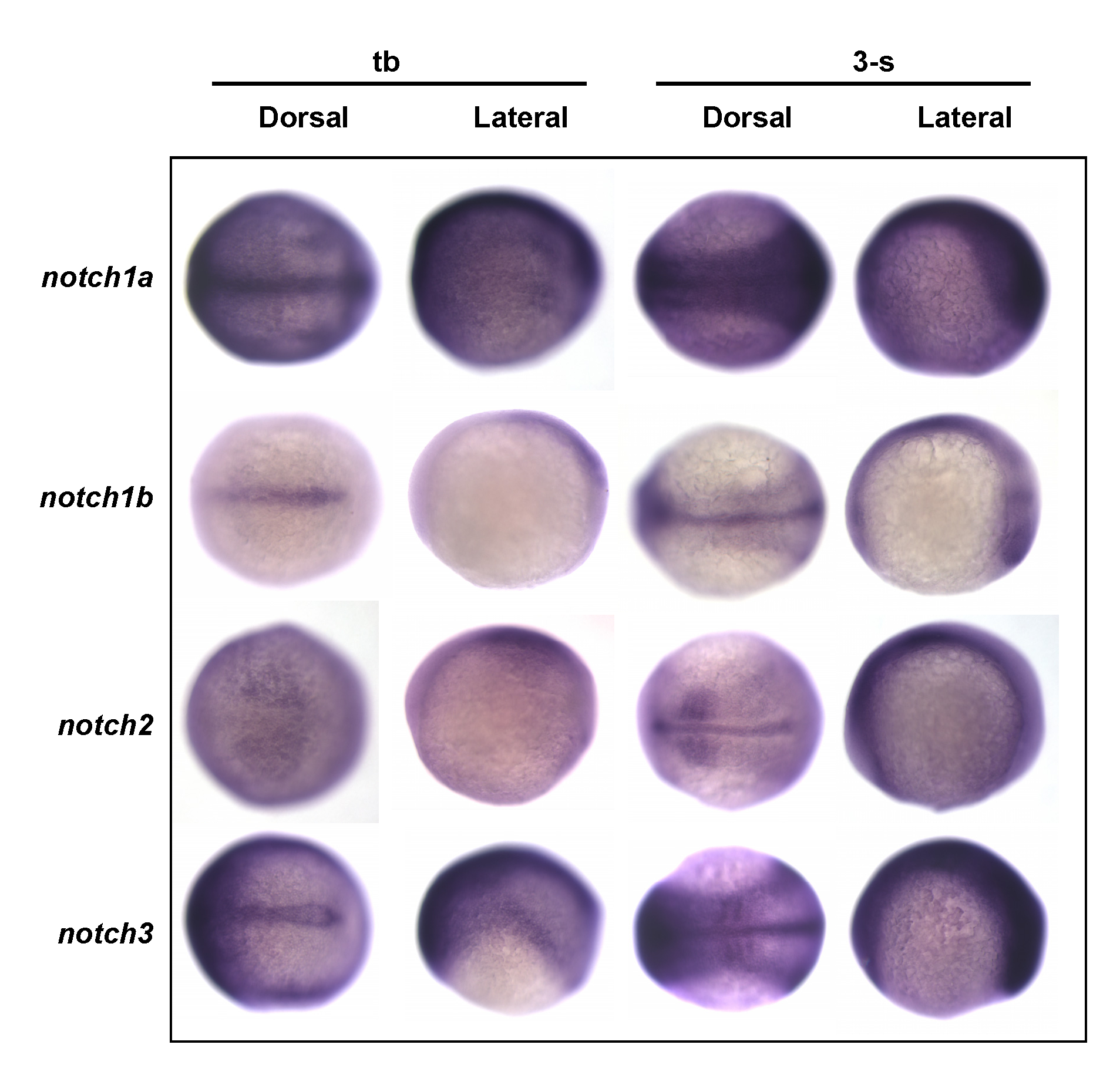

Supplement: Figure S3 — Screening for possible notch receptor expression in the epidermal ionocyte domain. Embryos aged at the tail bud (tb) to the 3-somite (3-s) stages were evaluated in situ with either (A) notch1a, (B) notch1b, (C) notch2, or (D) notch3 probes. Results show that notch1a was strongly and ubiquitously expressed in the epidermal ionocyte domain. Other notch genes, on the contrary, were expressed in the ventral ectoderm at a low level. (6.65 MB TIF) [file pone.0000302.s003.tif]

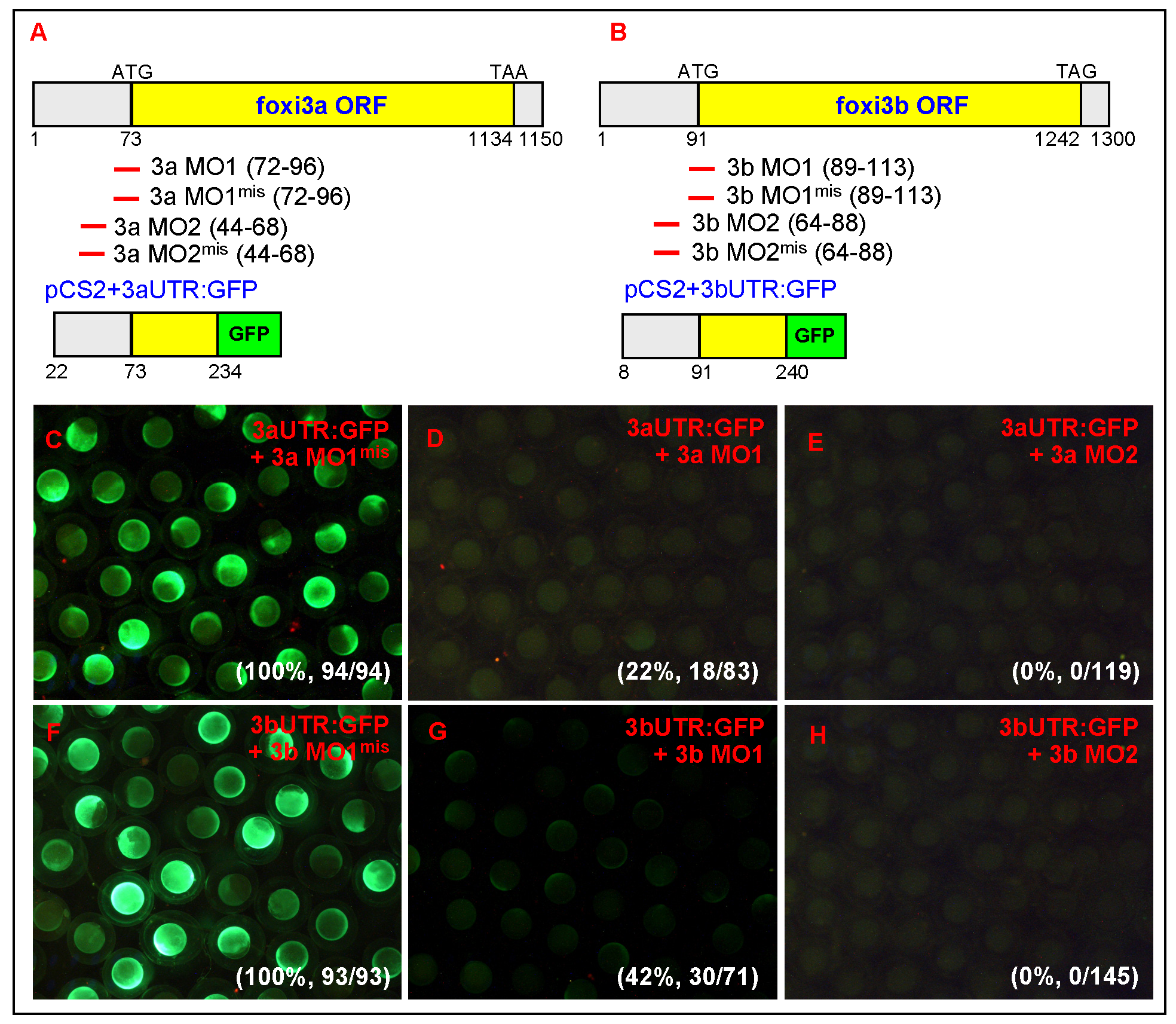

Supplement: Figure S4 — Control experiments to validate the specificity and efficacy of the morpholino. Schematic diagrams show the relative positions of the designed MOs and reporter constructs for either foxi3a (A) or foxi3b (B). The 5′UTR and partial exon 1 sequences of foxi3a or foxi3b were PCR-amplified from zebrafish cDNA and in-frame-fused with the green fluorescent protein (GFP) reporter gene. The resulting chimeric vectors of pCS2+3aUTR:GFP or pCS2+3bUTR:GFP contained the complementary sequences for testing the specificity and efficacy of both MO1 and MO2. (C) When five mismatched controls of 3a MO1mis (0.5 mM) were co-injected with 3aUTR:GFP mRNA (250 pg), all embryos (100%, n = 94) showed strong GFP expression. This result suggests that the 3a MO1mis is unable to target endogenous foxi3a mRNA. (D) When the 3a MO1 (0.5 mM) was co-injected with 3aUTR:GFP mRNA (250 pg), GFP expression was completely abolished in 78% of the injected embryos (n = 83). (E) When 3a MO2 (0.5 mM) was co-injected with 3aUTR:GFP mRNA (250 pg), GFP expression was completely abolished in all injected embryos (n = 119). This result suggests that both 3a MO1 and 3a MO2 can target endogenous foxi3a mRNA. However, the efficacy of 3a MO2 was superior to 3a MO1. (F) When five mismatched controls of 3b MO1mis (0.5 mM) were co-injected with 3bUTR:GFP mRNA (250 pg), all embryos (100%, n = 94) showed strong GFP expression. This result suggests that the 3b MO1mis is unable to target endogenous foxi3b mRNA. (G) When the 3b MO1 (0.5 mM) was co-injected with 3bUTR:GFP mRNA (250 pg), it was only sufficient to abolish GFP expression in 58% of injected embryos (n = 71). (E) When the 3b MO2 (0.5 mM) was co-injected with 3bUTR:GFP mRNA (250 pg), it was sufficient to abolish GFP expression in all injected embryos (n = 145). This result suggests that both 3b MO1 and 3b MO2 can target endogenous foxi3b mRNA. However, the efficacy of 3b MO2 was superior to 3b MO1. The number and percentage on the right bottom corner of C-H refer t [file pone.0000302.s004.tif]

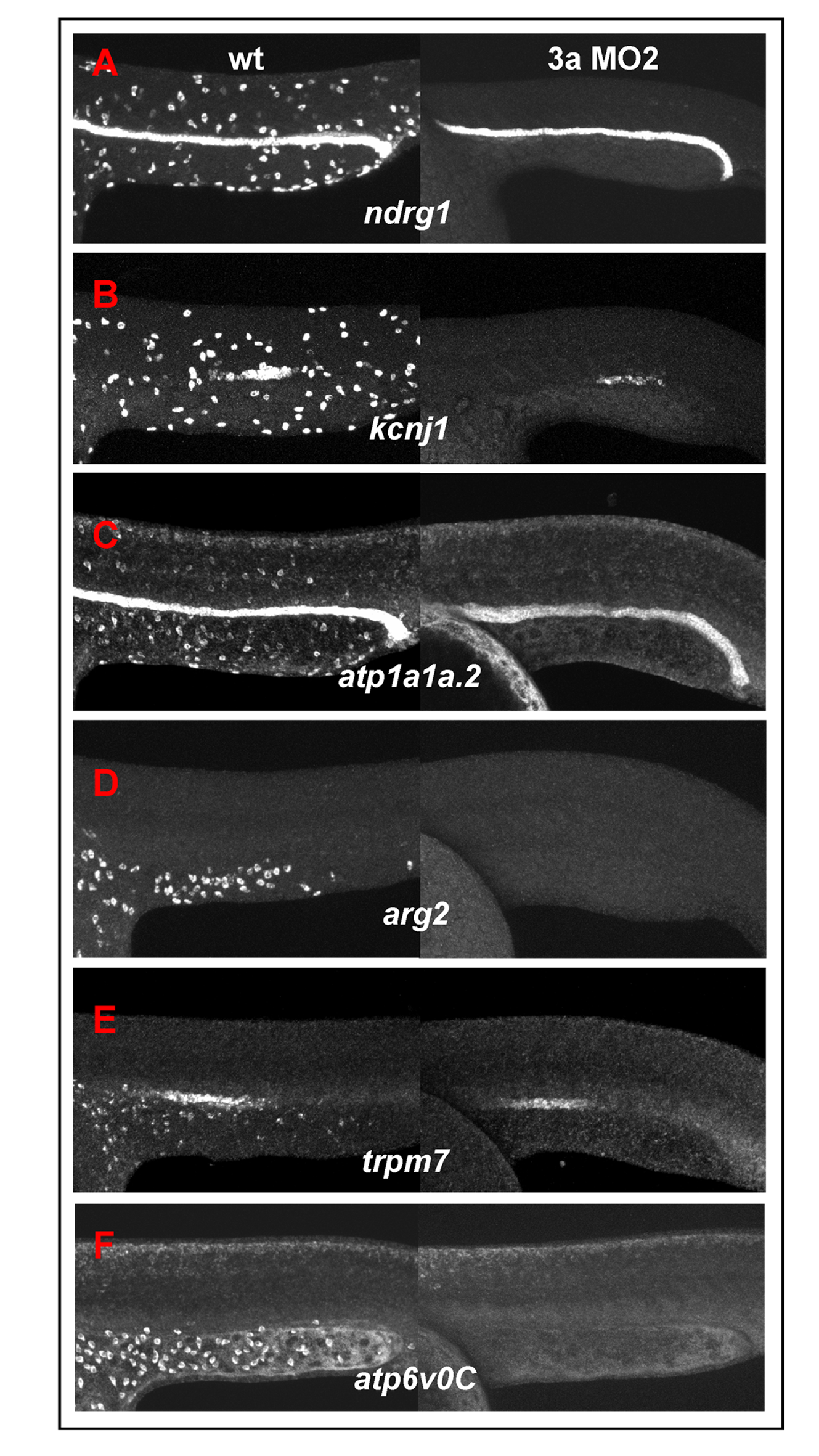

Supplement: Figure S5 — Global down-regulation of epidermal ionocyte markers in foxi3a morphants. (A-F) Comparison of the epidermal ionocyte marker expression between wild-type (left panel) and foxi3a morphants (right panel). The expression of markers is completely abolished in the epidermal ionocyte lineage in foxi3a morphants. Note that the pronephric duct expressions in ndrg1, kcnj1, atp1a1a.2 and trpm7 were largely undisturbed, which shows that the foxi3a morphant phenotype specifically targets epidermal ionocytes. Na+,K+-ATPase-rich cell (NaRC) markers were ndrg1 (A), kcnj1 (B), and atp1a1a.2 (C). H+-ATPase-rich cell (HRC) markers were arg2 (D), trpm7 (E), and atp6voC (F). All embryos were scored at 24 hours post-fertilization (hpf). (9.54 MB TIF) [file pone.0000302.s005.tif]

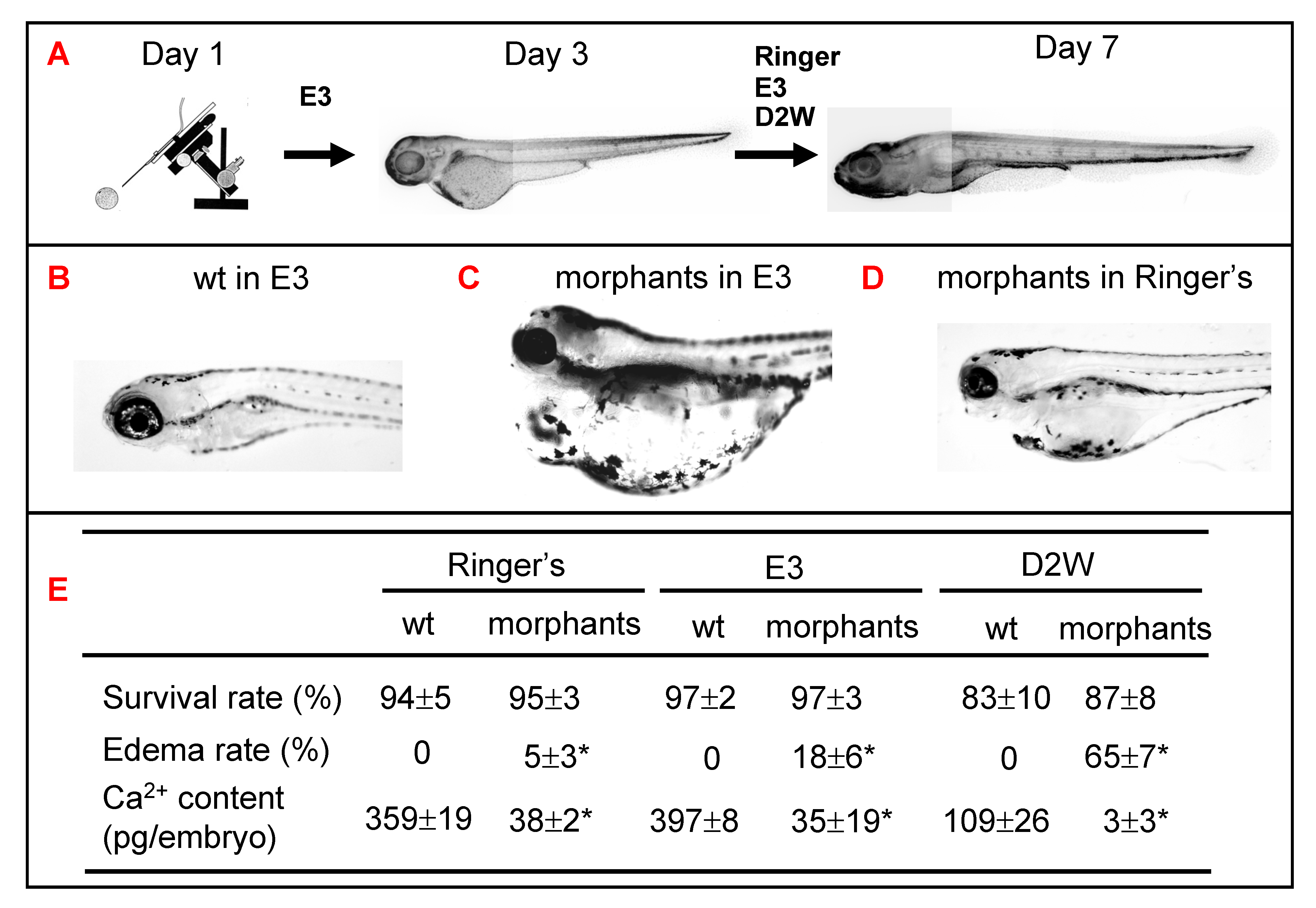

Supplement: Figure S6 — Role of epidermal ionocytes in water and ion homeostasis in zebrafish embryos. (A) Procedure to assay the physiological functions of epidermal ionocytes in zebrafish embryos. After injecting them with foxi3a MO, morphants were initially raised in E3 up to 3 days post-fertilization (dpf) and then challenged with either Ringer's solution, E3, or double-distilled water (D2W). The survival rate, edema rate, and whole-body Ca2+ content between wild-types (wt) and morphants were measured at 7 dpf, and results are summarized in (E). The wild-types had a strong water balance ability and showed no edema phenotype in either E3 (B), Ringer's solution, or D2W (not shown). The morphants displayed a severely edematous phenotype in hypotonic E3 (C) or D2W (not shown), while the abnormality was greatly rescued in isotonic Ringer's solution (D). The values are shown as the mean±SD (n = 10). Asterisks (*) indicate a significant difference from the wild-type (Student's t-test, p<0.05). (7.09 MB TIF) [file pone.0000302.s006.tif]
